# The effects of a digital health intervention on patient activation in chronic kidney disease

**DOI:** 10.1038/s41746-024-01296-1

**Published:** 2024-11-12

**Authors:** Courtney J. Lightfoot, Thomas J. Wilkinson, Gurneet K. Sohansoha, Clare L. Gillies, Noemi Vadaszy, Ella C. Ford, Melanie J. Davies, Thomas Yates, Alice C. Smith, Matthew P. M. Graham-Brown, On behalf of SMILE-K collaborators, Kieran McCafferty, Thomas Phillips, Barbara Winter-Goodwin, Siva Sridharan, Timothy Doulton, Carlito Adan, Kate Bramham, Aimun Ahmed, Andrew Nixon, Chris Goldsmith, Matthew Howse, Sandip Mitra, Sumith Abeygunasekara, Gowrie Balasubramaniam, Georgia Winnett, Sohail Ahmed, Rosie Donne, Sarah Brand, Robert Lewis, Nicholas Sangala, Steve Dickinson, Maarten Taal, Coralie Bingham, Simon Curran, Joyce Popoola, Andrew Stein, Kristin Veighey, Annika Wallis, Paul Laboi, Vicky Robins

**Affiliations:** 1 Leicester Kidney Lifestyle Team, Department of Population Health Sciences, University of Leicester, Leicester, UK; 2 NIHR Leicester Biomedical Research Centre, Leicester, UK; 3 Diabetes Research Centre, University of Leicester, Leicester, UK; 4 Leicester Real World Evidence Unit, Leicester Diabetes Centre, Leicester, UK; 5 Department of Cardiovascular Sciences, University of Leicester, Leicester, UK; 6 Department of Renal Medicine, University Hospitals of Leicester NHS Trust, Leicester, UK; 7 Barts Health NHS Trust, London, UK; 8 Dorset County Hospital NHS Foundation Trust, Dorset, UK; 9 East and North Hertfordshire NHS Trust, Stevenage, UK; 10 East Kent Hospitals University NHS Foundation Trust, Kent, UK; 11 King’s College Hospital NHS Foundation Trust, London, UK; 12 Lancashire Teaching Hospitals NHS Trust, Preston, UK; 13 Liverpool University Hospitals Foundation Trust, Liverpool, UK; 14 Manchester University NHS Foundation Trust, Manchester, UK; 15 Mid & South Essex NHS Foundation Trust, Essex, UK; 16 Northampton General Hospital NHS Trust, Northampton, UK; 17 Northern Care Alliance NHS Foundation Trust, Salford, UK; 18 Nottingham University Hospitals Trust, Nottingham, UK; 19 Portsmouth Hospitals University NHS Trust, Portsmouth, UK; 20 Royal Cornwall Hospitals NHS Trust, Cornwall, UK; 21 Royal Derby Hospitals NHS Trust, Derby, UK; 22 Royal Devon & Exeter NHS Foundation Trust, Exeter, UK; 23 Sheffield Teaching Hospitals NHS Foundation Trust, Sheffield, UK; 24 St Georges University Hospitals NHS Foundation, London, UK; 25 University Hospitals Coventry and Warwickshire NHS Trust, Coventry, UK; 26 University Hospital Southampton NHS Foundation Trust, Southampton, UK; 27 West Suffolk NHS Foundation Trust, Suffolk, UK; 28 York & Scarborough Teaching Hospital NHS Foundation Trust, York, UK

**Keywords:** Patient education, Health care

## Abstract

My Kidneys & Me (MK&M), a digital health intervention delivering specialist health and lifestyle education for people with CKD, was developed and its effects tested (SMILE-K trial, ISRCTN18314195, 18/12/2020). 420 adult patients with CKD stages 3–4 were recruited and randomised 2:1 to intervention (MK&M) (*n* = 280) or control (*n* = 140) groups. Outcomes, including Patient Activation Measure (PAM-13), were collected at baseline and 20 weeks. Complete case (CC) and per-protocol (PP) analyses were conducted. 210 (75%) participants used MK&M more than once. PAM-13 increased at 20 weeks compared to control (CC: +3.1 (95%CI: −0.2 to 6.4), *P* = 0.065; PP: +3.6 (95%CI: 0.2 to 7.0), *P* = 0.041). In those with low activation at baseline, significant between-group differences were observed (CC: +6.6 (95%CI: 1.3 to 11.9), *P* = 0.016; PP: +9.2 (95%CI: 4.0 to 14.6), *P* < 0.001) favouring MK&M group. MK&M improved patient activation in those who used the resource compared to standard care, although the overall effect was non-significant. The greatest benefits were seen in those with low activation.

## Introduction

Chronic kidney disease (CKD) affects ~10% of the global population^1^. By 2040 it is projected to be the fifth leading cause of death globally^2^. Most people living with CKD have early-stage disease, and are not managed by specialist kidney teams^3^. Whilst the risk of developing end-stage kidney disease (ESKD) is low, even early-stage CKD is associated with significant morbidity, disease burden and excess mortality^4^. Individuals with early-stage CKD have limited awareness of their condition and its implications compared to those with more advanced CKD^5,6^. Raising patient awareness of CKD and its complications is essential for optimal management to prevent disease progression and associated health complications^7^.

The importance of self-management to improve outcomes for people with long-term health conditions was highlighted in the National Health Service (NHS) Long Term Plan^8^ and is reflected in the National Institute for Health and Care Excellence (NICE) guidelines for CKD^9^. To effectively self-manage, individuals need to have relevant disease-specific knowledge to understand what to do and why, skills to perform required tasks or behaviours, and the confidence to perform them^10^ – this is termed patient activation^11^. For people with CKD, self-management encompasses a range of behaviours from medication adherence and symptom monitoring to lifestyle modifications and learning to live with the emotional consequences^10^. Improving patient awareness and knowledge of CKD is an essential part of strategies to successfully implement interventions that slow progression to ESKD and improve cardiovascular and health outcomes^7^. Economic modelling shows that improved implementation of kidney-related healthcare interventions, including improved CKD management, could save >10,000 lives and ~50,000 quality-adjusted life years in the UK over the next decade^2^.

In the last decade, there have been major advances in mobile technology which offer potential solutions to deliver healthcare digitally. The development of digital health interventions (DHIs) to support self-management and health-promoting behaviours was accelerated by the COVID-19 pandemic. To support self-management in people with non-dialysis CKD, we co-developed ‘My Kidneys & Me’ (MK&M), an evidence- and theory-based DHI that provides tailored, interactive information and support to improve CKD awareness and understanding, health knowledge, health-promoting behaviours and confidence^12,13^. We hypothesised that MK&M would: 1. increase patient activation and 2. improve related self-management knowledge and behaviours.

## Results

### Participant characteristics

**Fig. 1:**
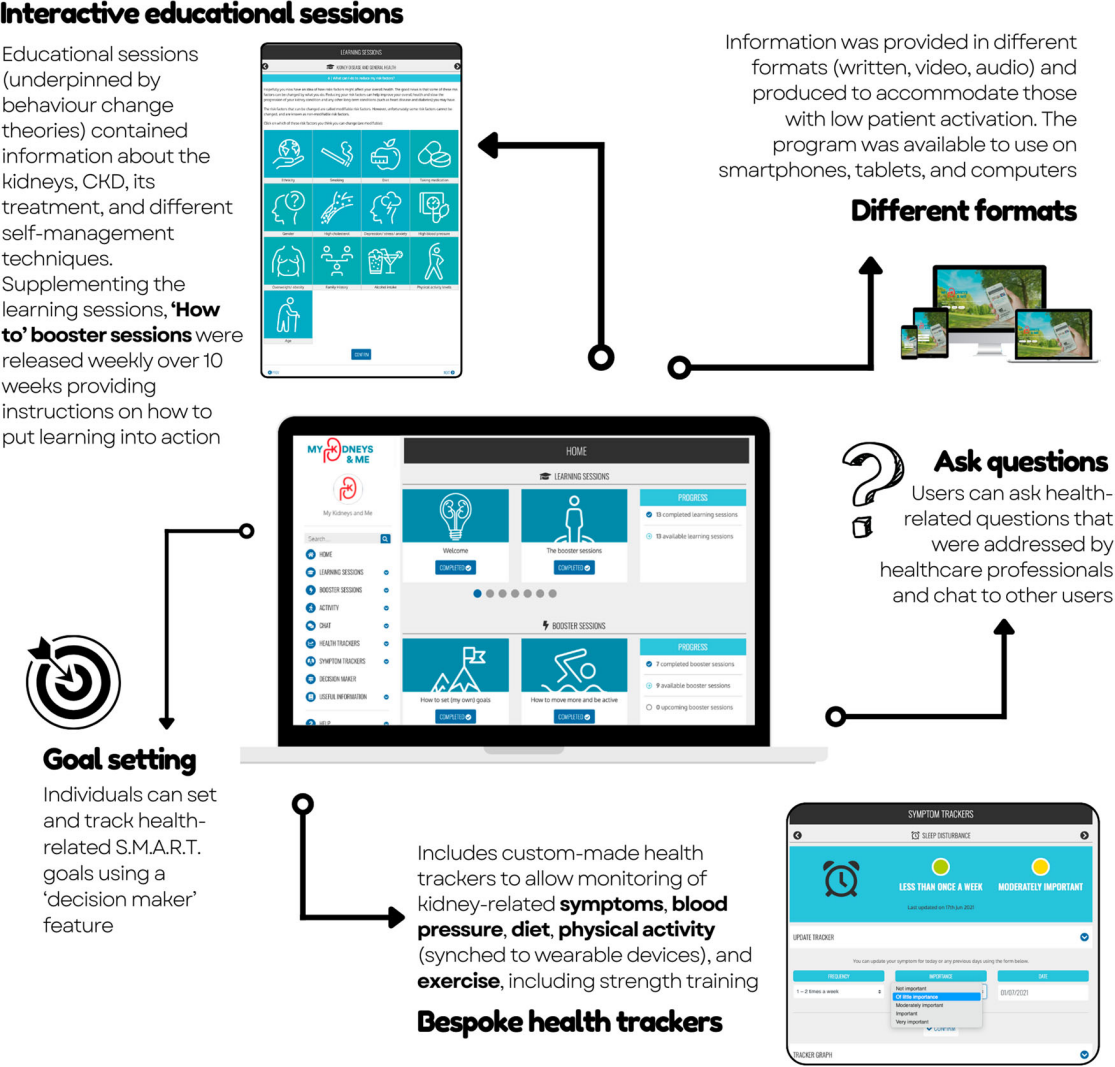
My Kidneys & Me dashboard and features. Illustration of the My Kidneys & Me dashboard and key features.

A total of 6802 potential participants were sent a study invitation. No follow-up approaches were made to these individuals. Of those invited, 875 expressed an interest in participation and were sent the participant information sheet. Of these, 533 consented to participate, and 420 completed the baseline measure and were randomised (intervention: *n* = 280; control: *n* = 140) and are included in the analyses (Fig. 1). The mean age of recruited participants was 59.8 (±13.4) years, 60% were male, 92% were White British and the mean eGFR was 38.8 (±18.8) ml/min/1.73 m^2^ (Table 1). No adverse events were observed.

**Table 1:** Participant characteristics

	All	Intervention	Control
	(*n* = 420)	(*n* = 280)	(*n* = 140)
Age, years	59.8 (±13.4)	59.4 (±13.6)	60.7 (±12.8)
Sex male, *n* (%)	250 (60%)	161 (58%)	89 (64%)
Ethnicity, *n* (%)			
White British	384 (92%)	254 (91%)	130 (93%)
Other White	14 (3%)	8 (3%)	6 (4%)
South Asian	10 (2%)	8 (3%)	2 (1%)
Black	5 (1%)	4 (1%)	1 (1%)
Other ethnic group	7 (2%)	5 (2%)	1 (1%)
Education, *n* (%)			
None	8 (2%)	4 (1%)	4 (3%)
Primary school	2 (1%)	1 (<1%)	1 (1%)
High school	82 (20%)	1 (<1%)	1 (1%)
College	133 (32%)	90 (33%)	43 (31%)
University	138 (33%)	87 (32%)	51 (37%)
Other trade/ vocation qualification	51 (12%)	39 (14%)	12 (9%)
Occupation, *n* (%)			
Retired	183 (45%)	115 (43%)	68 (50%)
Employed	208 (51%)	145 (54%)	63 (46%)
Unemployed	12 (3%)	6 (2%)	6 (4%)
Other (disabled, long-term sick leave)	4 (1%)	4 (1%)	0
Rurality, *n* (%)			
Rural	110 (26%)	70 (25%)	40 (29%)
Rural town and fringe	51 (12%)	32 (11%)	19 (14%)
Rural town and fringe in sparse setting	3 (<1%)	3 (1%)	0
Rural village and dispersed	52 (12%)	34 (12%)	18 (13%)
Rural village and dispersed in sparse setting	4 (1%)	1 (<1%)	3 (2%)
Urban	305 (72%)	208 (74%)	97 (69%)
Urban city and town	224 (53%)	155 (55%)	69 (49%)
Urban major conurbation	1 (<1%)	1 (<1%)	6 (4%)
Urban minor conurbation	14 (3%)	8 (3%)	22 (16%)
Multiple index of devprivation	6.4 (±2.6)	6.3 (±2.6)	6.7 (±2.5)
Blood pathology			
eGFR, mL/min/1.73m^2^	38.8 (±18.8)	38.4 (±17.1)	39.4 (±21.7)
Creatinine, mg/dl	184.2 (±74.3)	181.9 (±72.5)	188.7 (77.9)
Haemoglobin, mg/dl	127.7 (±19.0)	128.0 (±18.3)	127.1 (±20.4)
Albumin, mg/dl	40.1 (±5.8)	40.4 (±6.3)	39.7 (±4.7)
ACR, mg/g	58.4 (±92.0)	46.6 (±73.6)	74.7 (±112.3)
HbA1C, %	41.0 (±15.4)	41.7 (±15.7)	40.0 (±15.1)
Urea, mg/dl	12.7 (±8.1)	12.2 (±5.9)	13.6 (±11.1)
CRP, mg/L	10.4 (±27.7)	9.3 (±27.8)	12.3 (±27.8)
Systolic blood pressure, mm Hg	138.3 (±18.9)	138.9 (±19.2)	137.0 (±18.3)
Diastolic blood pressure, mm Hg	78.0 (±12.1)	78.5 (±11.3)	76.8 (±13.9)
Comorbidities			
Number of comorbidities	2.5 (±1.6)	2.5 (±1.5)	2.6 (±1.6)
Hypertension, *n* (%)	316 (77%)	209 (77%)	107 (78%)
Type 2 Diabetes, *n* (%)	81 (24%)	57 (25%)	24 (22%)
Heart problems, *n* (%)	89 (26%)	55 (24%)	34 (32%)
Blood vessel/circulation problems, *n* (%)	71 (22%)	49 (22%)	22 (21%)
Lung or breathing problems, *n* (%)	108 (32%)	71 (32%)	37 (33%)
Joint, bone or muscle problems, *n* (%)	158 (46%)	109 (47%)	49 (43%)
Depression, anxiety or other mental health problems, *n* (%)	101 (30%)	66 (30%)	35 (32%)
Body mass index, kg/m^2^	29.1 (±6.5)	29.4 (±7.1)	28.6 (±5.3)
PAM-13 score	61.9 (±14.9)	61.8 (±14.8)	62.1 (±14.9)
Low activation, *n* (%)	153 (36%)	100 (36%)	53 (38%)
CKD-SMKT – kidney health awareness	2.9 (±0.9)	2.9 (±0.9)	2.9 (±0.9)
CKD-SMKT – correct response	77.5 (±27.2)	78.1 (±26.6)	76.2 (±28.3)
CKD-SMKT – behaviours performed	5.4 (±1.9)	5.4 (±2.0)	5.3 (±1.8)
KSQ (frequency)	22.8 (±11.3)	22.7 (±11.6)	22.9 (±10.7)
UKDDQ	65.8 (±9.0)	65.8 (±9.2)	65.7 (±8.6)
MARS-5	23.6 (±2.1)	23.6 (±1.6)	23.6 (±1.9)
SARC-F	1.5 (±2.1)	1.6 (±2.1)	1.4 (±2.0)
STS-60	23.2 (±10.7)	23.2 (±11.2)	23.0 (±9.4)

Data presented as mean and SD, unless otherwise stated.

*eGFR* estimated glomerular filtration rate, *ARC* albumin to creatinine ratio (ACR), *CRP* C-reactive protein, *PAM-13* Patient Activation Measure, *CKD-SMKT* Chronic Kidney Disease – Self-Management Knowledge Tool, *KSQ* Kidney Symptom Questionnaire, *UKKDDQ* UK Diabetes and Diet Questionnaire, *MARS-5* Medication Adherence Rating Scale 5, *SARC-F* Strength, Assistance in walking, Rise from a chair, Climb stairs, and Falls, *STS-60* Sit-To-Stand 60.

### Intervention access and usage

The median number of logins per person over 20 weeks was 10.0 (IQR 4.0–28.0) (low activated: 9.0, 3.3–19.5; high activated: 11.0, 4.5–29.0). The median time per login was 12.6 mins (IQR 6.8–24.9 mins) (low activated: 14.1, 7.7–29.8; high activated: 11.5, 6.7–23.1). 70 participants in the intervention group did not activate their MK&M account or only logged in once, meaning *n* = 210 (75%) were included in the PP analyses (participant characteristics can be found in Supplementary Table 1).

### Primary outcome (PAM-13)

All particpants (*n* = 420) completed the PAM-13 at baseline, *n* = 282 (67%; *n* = 168 intervention, *n* = 114 control) at 10 weeks, and *n* = 224 (53%; *n* = 137 intervention, *n* = 87 control) at 20 weeks. Mean baseline PAM-13 score was 61.9 (±14.9) (intervention: 61.8 (±14.8); control: 62.1 (±14.9)). Participant characteristics of those who did and did not complete the primary outcome at the primary endpoint (20 weeks) can be found in Supplementary Table 2.

Changes in PAM-13 scores for CC and PP analyses are shown in Fig. 3a, b, respectively. In a CC analysis, no significant between-group differences were observed in PAM-13 at 20 weeks (±3.1 (95%CI: −0.2 to 6.4), *P* = 0.065). The change for PAM-13 score between baseline and 20 weeks in the intervention group was +4.1 (95%CI: 2.2 to 6.8) and for the control group was +1.0 (95% CI: −1.6 to 3.6) (Table 2). In the PP analysis, a significant between group difference was observed in PAM-13 score at 20 weeks ( ± 3.6 (95%CI: 0.2 to 7.0), *P* = 0.041). Between baseline and 20 weeks, a mean increase of 4.5 points (95%CI: 2.2 to 7.0) was observed in the intervention group (Table 3). No significant between group differences were observed at 10 weeks (CC: ±1.5 (95%CI: −1.4 to 4.5), PP: ±2.2 (95%CI: −1.0 to 5.4)). The change for PAM-13 score between baseline and 10 weeks in the intervention group was +3.0 (95%CI: 1.12 to 4.8) (PP: +3.6 (95%CI: 1.1 to 4.8)) and for the control group was +1.5 (95% CI: −0.8 to 3.7) (PP: +1.4 (95%CI: −0.9 to 3.5)).

**Fig. 2:**
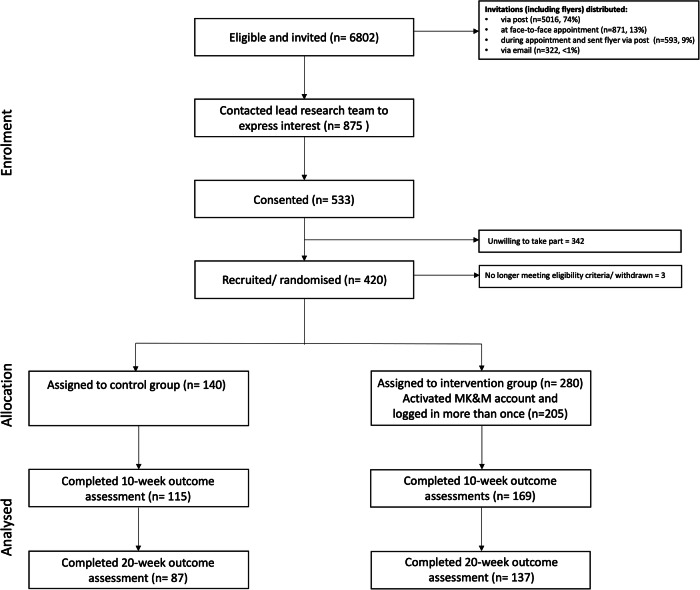
CONSORT diagram. Flowchart depicting progress through the trial phases.

**Table 2:** Changes in primary and secondary outcomes from baseline to week 10 and week 20: results from a complete case analysis

	Number of participants		Mean change from baseline (95% CI)		Mean intervention effect (95% CI)^a^	*P* value
	Control	Intervention	Control	Intervention		
Primary outcome						
PAM-13						
Week 10	114	168	1.47 (95% CI: −0.81 to 3.74)	2.97 (95% CI: 1.10 to 4.84)	1.50 (95% CI: −1.44 to 4.45)	0.316
Week 20	87	137	0.92 (95% CI: −1.61 to 3.45)	4.12 (95% CI: 2.20 to 6.81)	3.10 (95% CI: −0.19 to 6.39)	0.065
Secondary outcomes						
CKD-SMKT – kidney health awareness						
Week 10	113	168	−0.35 (95% CI: −0.47 to −0.22)	−0.45 (95% CI: -0.34 to -0.55)	−0.10 (95% CI: −0.26 to −0.06)	0.225
Week 20	86	136	−0.35 (95% CI: −0.50 to −0.21)	−0.52 (95% CI: −0.63 to −0.40)	−0.16 (95% CI: −0.35 to 0.02)	0.083
CKD-SMKT – correct response						
Week 10	114	168	5.82 (95% CI: 1.8 to –9.77)	9.65 (95% CI: 6.39 to 12.90)	3.83 (95% CI: −1.29 to 8.95)	0.142
Week 20	87	137	7.20 (95% CI: 2.06 to 12.34)	7.43 (95% CI: 3.34 to 11.52)	0.23 (95% CI: −6.34 to 6.80)	0.945
CKD-SMKT – behaviours performed						
Week 10	98	142	−0.66 (95% CI: −0.94 to −0.38)	−0.38 (95 CI: −0.62 to −0.15)	0.28 (95% CI: −0.09 to 0.64)	0.135
Week 20	73	111	0.28 (95% CI: 0.06 to 0.61)	0.57 (95% CI: 0.30 to 0.84)	0.30 (95%CI: -0.13 to 0.73)	0.176
KSQ (frequency)						
Week 10	113	168	0.19 (95% CI: −0.86 to 1.23)	0.28 (95% CI: −0.58 to 1.14)	0.09 (95% CI: −1.26 to 1.45)	0.893
Week 20	87	137	0.79 (95% CI: −0.47 to 2.06)	−0.52 (95% CI: −1.53 to 0.49)	−1.31 (95% CI: −2.93 to 0.31)	0.111
UKDDQ						
Week 10	113	168	−2.46 (95% CI: −3.58 to −1.34)	−1.18 (95% CI: −2.10 to −0.26)	1.28 (95% CI: −0.17 to 2.73)	0.083
Week 20	87	137	0.70 (95% CI: −0.53 to 1.93)	2.51 (95% CI: 1.53 to 3.49)	1.81 (95% CI: 0.24 to 3.38)	**0.024**
MARS-5						
Week 10	110	160	0.02 (95% CI: −0.28 to 0.33)	−0.15 (95% CI: −0.41 to 0.10)	−0.18 (95% CI: −0.57 to 0.21)	0.371
Week 20	84	129	−0.12 (95% CI: −0.45 to 0.21)	0.06 (95% CI: −0.20 to 0.33)	0.18 (95% CI: −0.24 to 0.61)	0.391
SARC-F						
Week 10	114	168	0.09 (95% CI: −0.12 to 0.29)	0.07 (95% CI: −0.09 to 0.24)	−0.01 (95% CI: −0.27 to 0.25)	0.933
Week 20	87	137	0.19 (95% CI: −0.01 to 0.40)	−0.10 (95% CI: −0.27 to 0.07)	−0.29 (95% CI: −0.56 to −0.18)	**0.037**
STS-60						
Week 10	98	149	0.40 (95% CI: −0.75 to 1.54)	1.27 (95% CI: 0.34 to 2.20)	0.87 (95% CI: −0.60 to 2.35)	0.243
Week 20	77	117	0.68 (95% CI: −0.99 to 2.35)	2.76 (95% CI: 1.40 to 4.11)	2.07 (95% CI: −0.08 to 4.23)	0.059

*NB. PAM-13* Patient Activation Measure, *CKD-SMKT* Chronic Kidney Disease – Self-Management Knowledge Tool, *KSQ* Kidney Symptom Questionnaire, *UKKDDQ* UK Diabetes and Diet Questionnaire, *MARS-5* Medication Adherence Rating Scale 5, *SARC-F* Strength, Assistance in walking, Rise from a chair, Climb stairs, and Falls, *STS-60* Sit-To-Stand 60.

^a^Intervention value minus control value, adjusted for baseline value and age.

Bolded values are significant (*P* < 0.05).

**Table 3:** Changes in primary and secondary outcomes from baseline to week 10 and week 20: results from a per-protocol analysis

	Number of participants		Mean change from baseline (95% CI)		Mean intervention effect (95% CI)^a^	*P* value
	Control	Intervention	Control	Intervention		
Primary outcome						
PAM-13						
Week 10	114	123	1.47 (95% CI: −0.81 to 3.74)	3.58 (95% CI: 1.10 to 4.84)	2.22 (95% CI: −0.95 to 5.39)	0.169
Week 20	87	105	0.92 (95% CI: −1.61 to 3.45)	4.50 (95% CI: 2.20 to 6.81)	3.58 (95% CI: 0.16 to 7.00)	**0.041**
Secondary outcomes						
CKD-SMKT – kidney health awareness						
Week 10	113	123	−0.35 (95% CI: −0.47 to −0.23)	−0.54 (95% CI: −0.66 to −0.42)	−0.19 (95% CI: −0.37 to −0.02)	**0.026**
Week 20	86	104	−0.35 (95% CI: −0.50 to −0.20)	−0.60 (95% CI: −0.70 to −0.43)	−0.22 (95% CI: −0.42 to −0.02)	**0.030**
CKD-SMKT – correct response						
Week 10	114	123	5.95 (95% CI: 2.40 to 9.50)	12.72 (95% CI: 9.29 to 16.16)	6.77 (95% CI: 1.83 to 11.72)	**0.007**
Week 20	87	105	7.38 (95% CI: 2.15 to 12.62)	7.18 (95% CI: 2.41 to 11.94)	−0.21 (95% CI: −7.29 to 6.87)	0.954
CKD-SMKT – behaviours performed						
Week 10	98	105	−0.66 (95% CI: −0.29 to −0.96)	−0.45 (95% CI: −0.81 to −0.16)	0.21 (95% CI: −0.18 to 0.61)	0.285
Week 20	73	86	0.29 (95% CI: 0.06 to 0.61)	0.57 (95% CI: 0.27 to 0.86)	0.28 (95%CI: -0.16 to 0.71)	0.216
KSQ (frequency)						
Week 10	113	123	0.21 (95% CI: −0.86 to 1.27)	0.41 (95% CI: −0.61 to 1.43)	0.21 (95% CI: −1.27 to 1.68)	0.783
Week 20	87	105	0.88 (95% CI: −0.46 to 2.04)	−0.34 (95% CI: −1.51 to 0.83)	−1.22 (95% CI: −2.96 to 0.52)	0.168
UKDDQ						
Week 10	113	123	−2.53 (95% CI: −3.64 to −1.42)	−1.37 (95% CI: −2.43 to −0.30)	1.12 (95% CI: −0.37 to 2.71)	0.136
Week 20	87	104	0.61 (95% CI: −0.57 to 1.80)	2.57 (95% CI: 1.49 to 3.66)	1.96 (95% CI: 0.35 to 3.57)	**0.017**
MARS-5						
Week 10	110	116	0.02 (95% CI: −0.25 to 0.29)	−0.08 (95% CI: −0.35 to 0.18)	−0.10 (95% CI: −0.48 to −0.28)	0.613
Week 20	84	99	−0.16 (95% CI: −0.49 to 0.18)	−0.02 (95% CI: −0.33 to 0.29)	0.14 (95% CI: −0.31 to 0.59)	0.543
SARC-F						
Week 10	114	123	0.09 (95% CI: −0.10 to 0.28)	0.02 (95% CI: −0.17 to 0.21)	−0.07 (95% CI: −0.34 to 0.20)	0.612
Week 20	87	105	0.20 (95% CI: −0.01 to 0.40)	−0.08 (95% CI: −0.27 to 0.11)	−0.27 (95% CI: −0.55 to 0.01)	0.057
STS-60						
Week 10	98	108	0.38 (95% CI: −0.76 to 1.52)	1.52 (95% CI: 0.44 to 2.61)	1.14 (95% CI: −0.44 to 2.72)	0.155
Week 20	77	90	0.69 (95% CI: −0.77 to 2.15)	3.12 (95% CI: 1.77 to 4.47)	2.43 (95% CI: 0.43 to 4.43)	**0.018**

*NB. PAM-13* Patient Activation Measure, *CKD-SMKT* Chronic Kidney Disease – Self-Management Knowledge Tool, *KSQ* Kidney Symptom Questionnaire, *UKKDDQ* UK Diabetes and Diet Questionnaire, *MARS-5* Medication Adherence Rating Scale 5, *SARC-F* Strength, Assistance in walking, Rise from a chair, Climb stairs, and Falls, *STS-60* Sit-To-Stand 60.

^a^Intervention value minus control value, adjusted for baseline value and age.

Bolded values are significant (*P* < 0.05).

#### Changes in PAM-13 between low and high baseline activation

Changes in PAM-13 scores for participants with baseline low and high activation are shown in Fig. 4a,b (data displayed in Supplementary Tables 3 and 4). There was a significant treatment group by activation level interaction (*P* < 0.001). In both CC (+6.6 (95%CI: 1.3 to 11.9), *P* = 0.016) and PP (+9.2 (95%CI: 4.0 to 14.6), *P* < 0.001) analyses, there were significant between-group differences from baseline to 20 weeks in PAM-13 in those with low activation at baseline. No significant between-group differences were observed for participants with high activation at baseline. Changes in PAM-13 levels from baseline to 20 weeks for both groups are displayed in Supplementary Fig. 1. Of those with low activation at baseline, 16% (*n* = 22) of intervention and 8% (*n* = 7) of control group moved from low levels of activation at baseline to high levels of activation at 20 weeks. There was no immediate relationship between time spent on the programme and change in patient activation.

**Fig. 3:**
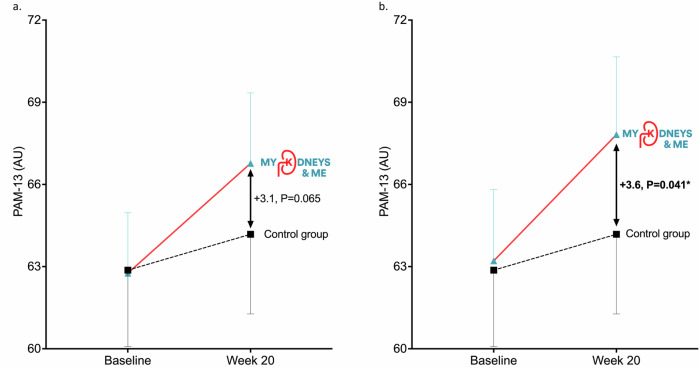
Changes in PAM-13 score from baseline to week 20. **a** CC analysis - includes all participants who completed PAM-13. **b** PP analysis - includes those who logged in, created an account and used the programme more than once. Data is shown as mean and standard deviation.

#### Changes in PAM-13 using MMRM (sensitivity analysis)

A total of 312 (intervention: 191; control: 121) participants were avalaible for MMRM analyses. The mean change in PAM-13 at 20 weeks was +4.1 and +1.1 in the intervention and control groups, respectively (study average treatment difference: +3.0, (95%CI: -0.3 to 6.2, *P* = 0.073) (Supplementary Table 5).

### Secondary outcomes

10-week and 20-week changes in secondary outcomes are displayed in Tables 2 and 3.

#### Self-management knowledge and behaviours

In CC analysis, no between-group differences were observed in perceived kidney health awareness scores (CKD-SMKT) at 10 weeks (*P* = 0.225) or 20 weeks (*P* = 0.083), but a significant between-group difference was observed at both 10 weeks (±0.2 (95%CI: -0.4 to 0.0), *P* = 0.026) and 20 weeks (±0.2 (95%CI: 0.0 to 0.4), *P* = 0.030) on PP analysis. There were no between-group differences found in the percentage of correct responses to the CKD-SMKT at 20 weeks (*P* = 0.945), but a significant difference was observed at 10 weeks (±6.8 (95%CI: 1.8 to 11.7), *P* = 0.007). No differences in the number of self-reported self-management behaviours performed were observed between groups (10 weeks: *P* = 0.135, 20 weeks: *P* = 0.176). A greater proportion of the intervention group reported improvements in CKD-SMKT compared to the control group at 20 weeks in both CC (52% vs 35%, *P* = 0.025) and PP analyses (54% vs 36% *P* = 0.014) (Table 4).

**Table 4:** Proportion reporting changes in secondary outcomes between baseline and 20 weeks

	Control (%)	Intervention (%)	*P* value
CKD SMKT - kidney health knowledge			0.052
* n*	86	136	–
Improvement	31 (36)	70 (52)	–
No change	49 (57)	55 (40)	–
Reduction	6 (7)	11 (8)	–
GPPAQ – level of activity			0.563
* n*	87	137	–
Improvement	6 (7)	5 (4)	–
No change	65 (75)	107 (78)	–
Reduction	16 (18)	25 (18)	–

Data presented as number (*n*) and percentage (%).

*P* value shows difference in proportions, significance recognised as *P* < 0.05.

*CKD-SMKT* Chronic Kidney Disease – Self-Management Knowledge Tool, *GPPAQ* GP Physical Activity Questionnaire.

#### Dietary behaviours

44% of MK&M (*n* = 122) and 40% of control (*n* = 55) group participants had a median UKDDQ score in the healthy range (mean: 3.3 ± 0.4) at baseline. This increased by 45% to 64% in MK&M participants (3.4 ± 0.4) and by 28% to 51% in control participants (3.4 ± 0.5) at 20 weeks. There were significant between-group differences observed for the change in total UKDDQ score at 20 weeks (CC: *P* = 0.024, PP: *P* = 0.017), but not at 10 weeks. The intervention group improved their total UKDDQ score between baseline and 20 weeks (CC: +2.5 (95%CI: 1.5 to 3.5), PP: +2.6 (95%CI: 1.5 to 3.7)). Significant decreases were observed between baseline and 10 weeks for control (CC: −2.5 (95%CI: −3.6 to −1.3, PP: −2.5 (95%CI: −3.6 to −1.4)) and intervention group (CC: −1.2 (95%CI: −2.1 to −0.3, PP: −1.4 (95%CI: −2.4 to −0.3)).

Significant differences between MK&M and the control group were observed at 20 weeks for healthy (±0.8 (95%CI: 0.1 to 1.5), *P* = 0.023) and less healthy dietary choices (±0.7 (95%CI: 0.1 to 1.3), *P* = 0.028) in CC analysis. The intervention group had significant increases in the number of healthy food choices (+0.7 (95%CI: 0.3 to 1.1) and decreases in the number of less healthy food choices (−0.7 (95%CI: −1.1 to −0.3)). The control group had a significant increase in the number of unhealthy food choices (+0.3 (95%CI: 0.0 to 0.6)).

#### Physical function

There was a significant between-group difference in SARC-F score at 20 weeks (CC: ±0.3 (95%CI: −0.6 to −0.2), *P* = 0.037). A significant difference was observed between groups in the number of completed repetitions in the STS-60 seconds at 20 weeks in PP analysis (±2.4 (95%CI: 0.4 to 4.4), *P* = 0.018). The intervention group significantly improved the number of completed repetitions in the STS-60 seconds by 10 weeks (CC: +1.3 (95%CI: 0.3 to 2.2), PP: +1.5 (95%CI: 0.4 to 2.6)) and 20 weeks (CC: +2.8 (95%CI: 1.4 to 4.1), PP: +3.1 (95%CI: 1.8 to 4.5)).

#### Symptom management

No significant differences were observed in the change of symptom frequency between groups. At 20 weeks, there was a numerical decrease in symptom frequency in the intervention group (CC: −0.5 (95%CI: −1.5 to 0.5)) and an increase in the control group (CC: +0.8 (95%CI: −0.5 to 2.1)), but no statistical within- or between-group differences.

#### Medication adherence

No significant changes in medication adherence (MARS-5) scores were observed at 10 weeks and 20 weeks. Both groups’ mean MARS-5 scores indicated poor medication adherence (score<25).

#### Physical activity

No significant differences in physical activity via the GPPAQ were observed (Table 4). Self-reported physical activity was low in both groups.

## Discussion

The SMILE-K trial did not demonstrate a statistically significant change in the primary endpoint, patient activation assessed by the PAM-13 score, at 20 weeks between participants assigned to receive the MK&M DHI and those who continued with usual care. Favourable effects of MK&M on PAM-13 were observed when only participants who registered and accessed MK&M were compared to the control group suggesting that, when used, MK&M leads to important improvements in patient activation and related CKD self-management behaviours. Moreover, efficacy was largest in patients with low patient activation at baseline. Whilst not effective in its current form when applied to all CKD patients, findings do suggest that in those who engage with MK&M and those with pre-existing low patient activation, MK&M is an appropriate means to deliver kidney health education to improve patient activation and self-management.

In the PP analysis, we observed a significant between-group difference in PAM-13 at 20 weeks favouring the intervention group. Whilst this difference of 3.6 fell short of the MCID of 4-points^14^ designed for general use, the absolute improvement in the intervention group was +4.5 in PP and +4.1 in CC analyses. We showed that those with low patient activation levels at baseline had profound increases in PAM-13 scores post-intervention. The between-group differences of +6.6 (CC) and +9.2 (PP) are comfortably above the MCID (in the general population) and almost half of the individuals who completed the intervention moved from PAM-13 Levels 1 or 2 (low activation) into 3 or 4 (high activation). These changes are similar to other research demonstrating that those with more passive roles in their healthcare show greater increases in PAM-13^15–18^. This is perhaps unsurprising given individuals with low activation have a greater need for self-management interventions, require more support to self-manage, and have more room for improvement. Subsequently, it is this group of patients in need of improvement where MK&M will be most effective. The high ceiling effect of PAM-13 in people living with CKD^19^ may explain why the change in PAM-13 between groups fell slightly short of the MCID; it may also be why a significant effect was observed in those with low baseline PAM. These findings highlight that MK&M is most relevant for those with low baseline activation, who are also those who are most in need of self-management education and support.

Increased CKD awareness and knowledge were observed in both groups, although greater changes were seen in the intervention group. Knowledge about a long-term condition and its treatment is an important component of self-management and patient activation^11^, thus increasing disease-related knowledge may explain the improvements seen in patient activation. Whilst raising CKD awareness is considered the first step to patient engagement and adherence^20,21^, it does not always translate to the performance of self-management behaviours^22^. We observed significant improvements in the performance of some of the related self-management behaviours in the intervention group, including dietary choices and physical function (subjective and objective). It may be that more time, beyond the study period, is needed to consolidate changes in other self-management behaviours, particularly those that may require stronger habit-formations, such as physical activity. Future work should evaluate the effects of the intervention with longer follow-up periods to assess the sustainability of changes in outcomes, as the study time-period has not captured the full effects of the intervention on self-management behaviours or health outcomes.

Individuals in this study had higher levels of patient activation than those reported by other studies^23–27^. There are several explanations for this. Firstly, the study recruited patients from secondary care CKD clinics who had already seen specialist teams and consequently likely had higher levels of CKD-related knowledge. The decision to run the study in secondary care was pragmatic and due to the difficulties of setting up the study in primary care during the COVID-19 pandemic. Secondly, the entirely remote nature of the study meant participation was highly dependent on participant motivation. Whilst the study design was remote out of necessity (owing to the COVID-19 pandemic), individuals with the lowest level of activation (Level 1) and health literacy may have been disadvantaged. The enrolment in the study was relatively low (13% of those initially invited), however, this figure represents the number of emails, letters or flyers sent or given to patients and many of these will not have been opened or read. There was no follow-up with patients to support participation and it is reasonable to suppose that follow-up and discussions would have improved participation. Indeed, participants were given no additional prompts or reminders to engage with the programme, and in clinical practice, prompts and reminders would be automated into clinical workflow to improve and sustain participation and engagement. The number of participants recruited from those who expressed interest in the study was good and in keeping with the proportions of patients recruited in the recently published Kidney BEAM trial^28^. Participants randomised to the MK&M intervention spent ~2.5 hours on MK&M, with some individuals accessing MK&M frequently and for longer periods. User engagement of MK&M is slightly greater than its diabetes counterpart MyDESMOND, with their participants logging in a median of 11 times, and spending a total of 105 minutes on the programme, over 6 months^29^. This suggests that whilst DHIs may not be appropriate for everyone, there is a need and appetite for DHIs amongst those with CKD. Many interconnecting factors affect an individual’s ability to engage with DHIs^30^ and work is planned to revise MK&M to improve accessibility and support more individuals to engage and interact with the platform.

As mentioned, this study was conducted in secondary care. Given that the majority of individuals with CKD are not managed in secondary care^31^, the real benefit for MK&M may be in those treated in primary care where people may have lower activation, have poorer awareness of CKD and its implications, and do not currently have access to relevant education and tools^5^. Early identification and intervention in CKD are key strategies to slow disease progression and improve cardiovascular and other health outcomes^32^. Raising awareness of CKD is paramount to optimise management^7^ and the provision of information about CKD and its management will positively impact an individual’s ability to self-manage their health. Providing MK&M in primary care will likely have the greatest impact given the evidence of successful self-management behaviours on delaying disease progression, preventing progression to ESKD, reducing cardiovascular morbidity and mortality, and other adverse health outcomes. Future work will assess the impact of resources like MK&M on these outcomes. Implementation of MK&M in clinical practice will also support the collaboration of shared knowledge and skills between primary and secondary care^33^ to ensure patients receive high-quality, person-centred care.

Unlike many self-management interventions, MK&M was not prescriptive and provided minimal interaction with a healthcare professional. Participants were provided access to MK&M and left to use the programme how they wished. Part of the SMILE-K trial involved qualitatively exploring the usability of MK&M and participants’ perspectives and experiences of using MK&M. These data will be used to further revise the programme to support uptake and engagement, particularly for underserved patient groups who stand to gain the most. Whether these revisions improve access for disadvantaged or underrepresented groups, and whether MK&M reduces referrals to secondary care, prevents unnecessary hospital admissions, and reduces healthcare costs, will be tested in future implementation studies in primary care settings and pathways. The programme is currently designed for those with non-dialysis CKD and mild to moderate stages of the condition, and thus is not appropriate for those with more advanced disease who are reaching ESKD. As the recommendations and decisions for those with advanced CKD reaching ESKD may be different than those with mild to moderate CKD, further work is required to make a similar programme to MK&M for those with more advanced CKD/ ESKD.

This study has several strengths and limitations. The main limitation is the amount of data that was lost to follow-up. At the primary end-point (20 weeks), there were 47% missing data, with greater proportions of missingness observed in the intervention group (51%) compared to control (38%). Several participants who completed the primary outcome did not complete some secondary outcomes, which may be a result of survey fatigue or perceived lack of relevance. The higher response rate observed in the control group compared to the intervention group may partly be explained by the provision of access to MK&M; the control group were given access to MK&M upon study competition. At 20 weeks, 52% participants still using the programme; however, 28% of those still using the programme did not complete the 20-week outcome assessment, suggesting lack of completion of the trial outcomes does not equate to lack of engagement with MK&M. Despite the large proportion of missing data, participant characteristics of those who completed the outcome measures were similar to those who did not, suggesting data was missing at random. Given the large proportion of missing data, methods of imputation were not appropriate due to the potential to introduce bias and inconsistency of effect estimates. To maintain good statistical properties under mild missing data assumptions (i.e. missing at random), MMRM has been recommended^34,35^. The use of MMRM^36^ has previously yielded confidence intervals with relatively good coverage accuracy and very low biases^37^. It is reassuring that the results observed from the MMRM analysis in this trial were similar to those observed in the CC analysis.

A key strength of the study design was the use of an internal pilot to determine the feasibility and acceptability of the trial design to inform the definitive trial^38^. Following the internal pilot phase, low completion rates were noted despite the continued use of the programme by participants. Consequently, a follow-up reminder email was introduced to attempt to increase the response rate and completion of the outcome assessments. Whilst the introduction of the reminder potentially improved the overall response rate, levels of 20 week outcome completion were still 53%. Having a more personalised approach, more patient contact, and additional options for completing outcome measures (such as written survey packs) may have increased outcome measure completion; however, this was not possible for this trial due to the pandemic. More work is needed to determine how to improve response rates for pragmatic decentralised clinical trials. Like others^17,39^, the effects of our intervention employed in this study may have been underestimated, given high activation scores at baseline. Comparing the groups by dividing them into low and high levels of activation highlighted the substantial benefit of the intervention for those with lower levels of activation. Reporting both CC and PP analyses, as per CONSORT guidelines^40^, gives an indication of the efficacy of the intervention when used, but the findings are not immediately generalisable or suggestive of wider effectiveness without careful revisions to the platform to improve engagement, particularly for those with lower health literacy and activation. Of those who completed the outcome measures, almost three-quarters of intervention participants were included in the PP analyses, demonstrating the efficacy of MK&M when used.

The findings have some additional generalisable limitations due to the large proportion of White participants (>90%), thus the findings may not be generalisable to those from minority ethnic groups and specific revision to improve engagement for these populations will be undertaken. Participants were well-educated and from higher socioeconomic groups which limits the generalisability of findings. Whilst an inability to speak English was not an exclusion criterion, communication, study information and MK&M, were provided in English, and thus individuals who could not speak, read, and/or write English were likely unable to participate. Due to the virtual nature of the study and MK&M programme, individuals with low levels of health and digital literacy were likely disadvantaged, and individuals without access to digital technology were ineligible. Trial processes that would ordinarily have engaged participants with lower health literacy and from a more diverse range of backgrounds were not possible due to the necessary remote design, and this is a limitation of the work. Further work is needed to improve the equity of decentralised clinical trials and digital health interventions for people with CKD from potentially disadvantaged groups. In addition, implementation of MK&M into routine practice is required to evaluate and understand the accessibility of MK&M in a real-life setting, without research barriers.

Overall, MK&M did not improve patient activation above that of usual care. However, increased patient activation and self-management behaviours were clearly demonstrated in 1) those who engaged with the programme and 2) those with low levels of pre-existing activation. As higher levels of patient activation are associated with better self-management and health outcomes and lower healthcare costs, MK&M has the potential to improve CKD healthcare management. Further work is needed to refine MK&M to increase uptake and engagement among disadvantaged and underserved groups.

## Methods

Findings are reported per the ‘CONSORT 2010 Statement’^40^ (Supplementary Table 6). The study was prospectively registered as ‘Self-Management Intervention through Lifestyle Education for Kidney health’ (SMILE-K) to the ISRCTN registry (ISRCTN18314195) on 18/12/2020. The study protocol is as previously published^13^.

### Study design and setting

The ‘Self-Management Intervention through Lifestyle Education for Kidney health’ (SMILE-K) trial was a 20 week prospective, multi-centre randomised-controlled, parallel-group trial (RCT) recruiting patients from 26 secondary care hospital sites across England, between May 2021 and December 2022. All study processes were conducted remotely due to the COVID-19 pandemic and for pragmatic evaluation. The intervention evaluated in this trial

The trial had an adaptive design with a mixed-method internal pilot to assess the feasibility and acceptability of conducting the definitive trial, allowing for data collection to continue whilst modifying the trial design based on the internal pilot findings. The internal pilot demonstrated that the design was feasible and acceptable^38^. The pilot identified areas for improvement (e.g., broadening the inclusion criteria, sending outcome assessment reminders) and the protocol was amended accordingly to improve the delivery of the SMILE-K trial (Supplementary Table 7).

### Ethics approval and consent to participate

This study was fully approved by the Research Ethics Committee-Leicester South on 19/11/2020 (reference: 17/EM/0357). All participants provided online informed consent. All participants were given the opportunity to ask questions throughout the consent process. The study was conducted in accordance with the Declaration of Helsinki. The trial is sponsored by the University of Leicester (rgo@le.ac.uk).

### Participants

Eligible participants were aged ≥18 years with CKD stages 3–4 (estimated glomerular filtration rate, eGFR 15–59 ml/min/1.73m^2^) not receiving renal replacement therapy or had an eGFR >60 ml/min/1.73m^2^ and were assessed by their nephrologist as having a kidney condition that was likely to progress, either because of the nature of their underlying kidney disease (e.g. polycystic kidney disease, relapsing glomerulonephritis), have high levels of proteinuria, or have co-morbidities that made risk of progression more likely (e.g. difficult to control blood pressure or reno-vascular disease). Eligible participants were invited via a letter and promotional flyer. Invitation methods (post, face-to-face, email) were decided by individual research sites. Following the initial invitation, interested participants contacted the lead research team via email. Interested participants were sent an online participant information sheet and consent form. Following consent, participants were sent a link to an online survey collecting baseline outcome measures.

### Randomisation

After completing baseline outcome measures, participants were randomised 2:1 into intervention or control groups using computer-generated randomisation^13^. Randomisation was stratified by age ( ≤63, >63 years) to ensure comparatively equal representative age characteristics in both groups. These values are based on the median age attained from preliminary unpublished data from two ongoing observational studies in nondialysis CKD patients by our group. Researchers at the lead site were not concealed to the allocation sequence and participants were not blinded to group allocation.

### Procedures

#### Intervention group: My Kidneys & Me

Participants randomised to the intervention group received immediate access to the MK&M programme with a unique user login. Hosted on the MyDESMOND platform^41^, a digital self-management programme adapted from the face-to-face Diabetes Education and Self-Management for Ongoing and Newly Diagnosed (DESMOND) programme^42^.

My Kidneys & Me is a systematically developed theory- and evidence-based digital self-management intervention for people with non-dialysis CKD; it is designed to provide holistic CKD-specific self-management education, support, and guidance for people living with mild to moderate CKD. Whilst MK&M provides in-depth information and support for people with early stages of CKD to manage their condition, the programme always encourages patients to check with their medical team if information provided in the programme seems contradictory to something they have been told by their healthcare team. The development of MK&M and its features are discussed in detail elsewhere^12,13^.

**Fig. 4:**
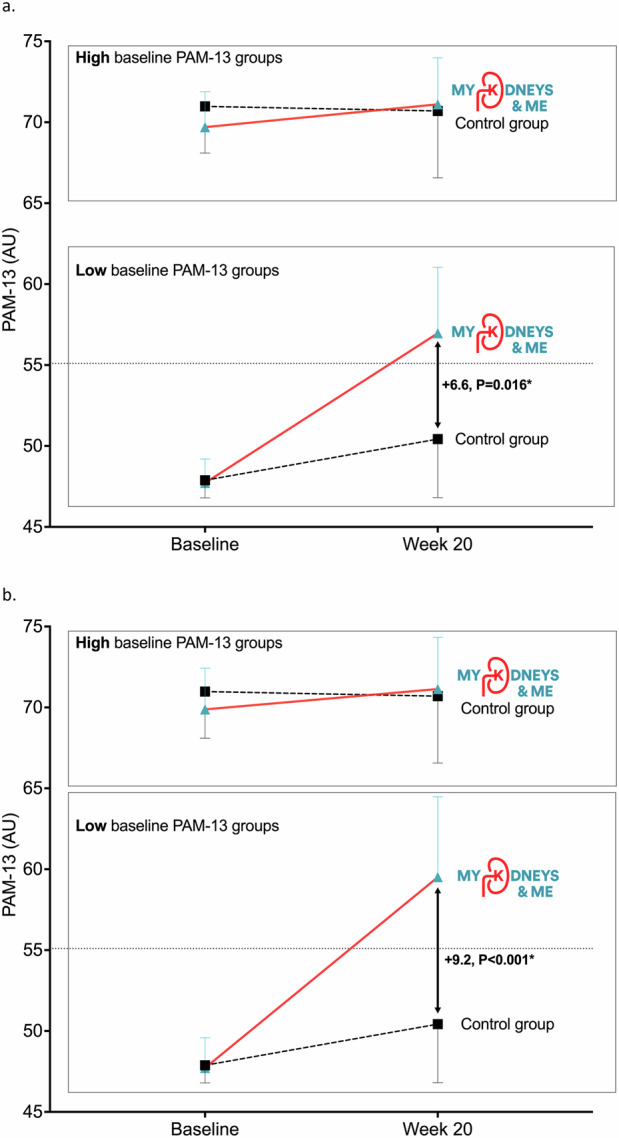
Changes in PAM-13 score from baseline to week 20 for participants with low and high baseline patient activation. **a** CC analysis - includes all participants who completed PAM-13. **b** PP analysis - includes those who logged in, created an account and used the programme more than once. Data shown as mean and standard deviation. High baseline group was defined as those with PAM-13 Levels 3 and 4, low baseline group was defined as those with PAM-13 Levels 1 and 2. Horizontal dashed lines indicate the cut-off between low and high activation.

The MK&M programme provided a structured 10 week programme with tailored, interactive information and support to improve: 1. patient activation: awareness and understanding of CKD, health-related knowledge, skills to engage in health-promoting behaviours, confidence in managing their health; 2. related CKD self-management knowledge and behaviours, including symptom management, lifestyle behaviours and physical function^12,13^(Fig. 4). This was achieved through the provision of content and materials designed to increase CKD knowledge and patient activation, reduce health risks, manage symptoms and improve physical function. Theories and behaviour change techniques selected include the Self-Management Framework^43^, Capability, Opportunity, Motivation Behaviour model components of the Behaviour Change Wheel and taxonomy of behaviour change techniques^44,45^, Health Action Process Approach Model^46^, Common Sense Model^47^ and Social Cognitive Theory^48^.

The programme components developed comprised educational and behaviour change sessions, health trackers (eg, monitoring blood pressure, symptoms and exercise), goal-setting features and forums for social support. As new content was released weekly for 10 weeks, 20 weeks was selected as the primary endpoint to allow participants time to review the educational content, put learning into action, and explore other features on the programme.

As part of the MK&M programme, participants received automated reminder emails when new content (i.e. the ‘How to’ sessions) was available to them. Participants also received automated reminder emails if they had not logged in after 7, 14 and 28 days.

#### Control group

The control group received usual care and were asked to follow their clinical care plans. All participants received access to MK&M following study completion.

### Outcome measures

Outcome measures were collected using Jisc Online Surveys (Bristol, UK) at baseline, 10 weeks and 20 weeks (primary endpoint).

### Sociodemographic

Sociodemographic data, including age, sex, ethnicity and social deprivation (postcode), were collected. Clinical data, including eGFR, aetiology of renal disease, other health conditions, haemoglobin and albumin, were extracted from medical records.

### Primary outcome

The primary outcome was the absolute change at 20 weeks in the Patient Activation Measure (PAM-13)^13^ score, a questionnaire that assesses an individual’s knowledge, skills and confidence in managing their health(care)^49^ and ability to effectively self-manage^10^. The PAM-13 is scored from 0-100; the score can be categorised into one of four levels: Level 1 ( ≤ 47; disengagement and disbelief), Level 2 (47.1–55.1; increasing awareness, confidence and knowledge), Level 3 (55.2-67; readiness and taking action), and Level 4 ( ≥ 67.1; sustainment). Levels 1 and 2 indicate low activation; Levels 3 and 4 indicate high activation. Advocated for use by NHS England, the PAM-13 is validated in CKD^19^.

### Secondary outcomes

Secondary outcomes assessed changes in key self-management knowledge and behaviours. These were the: Chronic Kidney Disease Self-Management Knowledge Tool (CKD-SMKT)^50^, Kidney Symptom Questionnaire (KSQ)^51^, Strength, assistance with walking, rising from a chair, climbing stairs and falls questionnaire (SARC-F)^52^, General Practice Physical Activity Questionnaire (GPPAQ)^53,54^, UK Diabetes and Diet Questionnaire (UKDDQ)^55^, Medication Adherence Report Scale (MARS-5)^56^ and Sit-To-Stand 60 (STS-60) (Full details in Supplementary Note 1). Usage statistics of MK&M were collected to inform the per-protocol (PP) analysis. Activation of their account and number of logins were used to determine participant engagement with MK&M. It was agreed that accessing and using MK&M more than once would require a level of engagement that could potentially influence the relationship between the DHI and primary outcome; thus, those who did not activate their MK&M account or only logged into the programme once were not included in the PP analysis.

### Sample size

An initial sample size of *n* = 432 participants, including dropouts, was estimated to detect a minimal clinical important difference (MCID) of 4-points in the PAM-13^13^. As these data were not specific to CKD, data from an internal pilot (pooled variance at 10 weeks: 283.798; estimated difference: 6.3^57^) was used to review the sample size accordingly^58,59^. Using previously published methodology^60,61^, this review yielded a revised minimum sample size of 151 (*β* = 0.90 and *α* = 0.050). Assuming an estimated 40% dropout (rate from internal pilot study was 38%), this provided a revised sample size of 211 (*n* = 141 in the intervention group, and *n* = 70 in the control group). Given the uncertainty in the variability of PAM-13 in CKD and the intention to identify an MCID for the PAM-13 in CKD using data from the full RCT if feasible^13^, it was agreed to continue recruiting until an a-priori end date (December 2022).

### Statistical analysis

Analyses were conducted as per the the prespecified statistical analysis plan (Supplementary Note 2). Unless otherwise stated, data are presented as mean ± standard deviation (SD), median and interquartile range (IQR (n-n)), or n (%). Change data are presented as means and 95% confidence intervals. Data were tested for normality using the Shapiro-Wilk test. The primary analysis used generalised linear regression models to test between-group differences for the primary and secondary outcomes. Models were adjusted for age and baseline value, with change between follow-up and baseline as the dependent variable and group assignment as a fixed factor. Analyses were conducted using both complete case (CC) (i.e. only participants with available data for outcomes of interest) and per-protocol (PP) approaches on both the primary and secondary outcomes. A PP analysis includes all randomised patients who met a specific minimum criteria^62^; in this case, the PP analysis excluded participants who did not activate their MK&M account or who only logged in once.

A subgroup analysis compared changes in the primary outcome measure at 20-week follow-up based on baseline ‘low’ or ‘high’ activation. Whether activation level modified the treatment effect was determined by the F-test significance when entered as an interaction in a generalised linear model, adjusting for age and baseline value. Additional sub-group analyses explored time spent on the programme and changes in patient activation.

A sensitivity analysis was conducted for the primary outcome at 20-week follow-up using a Mixed Model for Repeated Measures (MMRM)^36,37^. Under a MMRM approach, missing values are implicitly handled under the assumption of missing at random^36^. Fixed effects included group (intervention or control), timepoint (10 or 20 weeks), group by timepoint interaction, PAM-13 score at baseline, and age. An unstructured covariance matrix for measurements within the same participant was used. Data were analysed using SPSS 28 (IBM SPSS Statistics). Statistical significance was accepted as *P* < 0.05.

## Supplementary information

Supplementary Material

## Data Availability

All data are available in the main text or the Supplementary Information.

## Code availability

Analyses were conducted using IBM SPSS Statistics 28.0. The SPSS dataset and codes, containing de-identified data, and the SPSS syntax file used to generate the results in this paper are both available upon request and sent to the corresponding author.

## References

[1] GBD Chronic Kidney Disease Collaboration. Global, regional, and national burden of chronic kidney disease, 1990-2017: a systematic analysis for the Global Burden of Disease Study 2017. *Lancet* **395**, 709-733 (2020). 10.1016/S0140-6736(20)30045-3 PMC7049905 32061315

[2] Kidney Research UK. Kidney disease: A UK public health emergency. *The health economics of kidney disease to 2033*. (2023).

[3] Hull, S. A., Nitsch, D., Caplin, B., Griffith, K. & Wheeler, D. C. The National CKD Audit: a primary care condition that deserves more attention. *Br. J. Gen. Pract. : J. R. Coll. Gen. Practitioners* **68**, 356–357 (2018). 10.3399/bjgp18X697997 PMC6058639 30049752

[4] Kovesdy, C. P. Epidemiology of chronic kidney disease: an update 2022. *Kidney Int Suppl. (2011)* **12**, 7–11 (2022). 35529086 10.1016/j.kisu.2021.11.003 PMC9073222

[5] Greer, R. C., Crews, D. C. & Boulware, L. E. Challenges perceived by primary care providers to educating patients about chronic kidney disease. *J. Ren. Care* **38**, 174–181 (2012). 23176576 10.1111/j.1755-6686.2012.00323.x PMC3508733

[6] Tuot, D. S. et al. CKD awareness in the general population: performance of CKD-specific questions. *Kidney Med.* **1**, 43–50 (2019). 32734184 10.1016/j.xkme.2019.01.005 PMC7380399

[7] Evans, M. et al. A narrative review of chronic kidney disease in clinical practice: current challenges and future perspectives. *Adv. Ther.* **39**, 33–43 (2022). 34739697 10.1007/s12325-021-01927-z PMC8569052

[8] NHS England. *The NHS Long Term Plan* (2019).

[9] National Institute for Health and Care Excellence (NICE). *Chronic Kidney Disease: Assessment And Management* (2021). 34672500

[10] Lightfoot, C. J. et al. Patient activation: the cornerstone of effective self-management in chronic kidney disease? *Kidney Dialysis* **2**, 91–105 (2022). 37101653 10.3390/kidneydial2010012 PMC10127536

[11] Hibbard, J. H., Stockard, J., Mahoney, E. R. & Tusler, M. Development of the Patient Activation Measure (PAM): conceptualizing and measuring activation in patients and consumers. *Health Serv. Res.* **39**, 1005–1026 (2004). 15230939 10.1111/j.1475-6773.2004.00269.x PMC1361049

[12] Lightfoot, C. J. et al. The codevelopment of “My Kidneys & Me”: a digital self-management program for people with chronic kidney disease. *J. Med. Internet Res.* **24**, e39657 (2022). 36374538 10.2196/39657 PMC9706383

[13] Lightfoot, C. J., Wilkinson, T. J., Yates, T., Davies, M. J. & Smith, A. C. ‘Self-Management Intervention through Lifestyle Education for Kidney health’ (the SMILE-K study): protocol for a single-blind longitudinal randomised controlled trial with nested pilot study. *BMJ Open* **12**, e064916 (2022). 36385018 10.1136/bmjopen-2022-064916 PMC9670928

[14] Hibbard, J. H., Greene, J. & Tusler, M. Improving the outcomes of disease management by tailoring care to the patient’s level of activation. *Am. J. Manag Care* **15**, 353–360 (2009). 19514801

[15] Lindsay, A., Hibbard, J. H., Boothroyd, D. B., Glaseroff, A. & Asch, S. M. Patient activation changes as a potential signal for changes in health care costs: cohort study of US high-cost patients. *J. Gen. Intern. Med.* **33**, 2106–2112 (2018). 30291604 10.1007/s11606-018-4657-6 PMC6258627

[16] Miller, V. M. et al. Increasing patient activation through diabetes self-management education: Outcomes of DESMOND in regional Western Australia. *Patient Educ. Couns.* **103**, 848–853 (2020). 31676100 10.1016/j.pec.2019.10.013

[17] Shah, V. O. et al. A home-based educational intervention improves patient activation measures and diabetes health indicators among Zuni Indians. *PLoS ONE* **10**, e0125820 (2015). 25954817 10.1371/journal.pone.0125820 PMC4425648

[18] Deen, D., Lu, W.-H., Rothstein, D., Santana, L. & Gold, M. R. Asking questions: the effect of a brief intervention in community health centers on patient activation. *Patient Educ. Couns.* **84**, 257–260 (2011). 20800414 10.1016/j.pec.2010.07.026

[19] Lightfoot, C. J., Wilkinson, T. J., Memory, K. E., Palmer, J. & Smith, A. C. Reliability and validity of the patient activation measure in kidney disease: results of rasch analysis. *Clin. J. Am. Soc. Nephrol.* **16**, 880–888 (2021). 34117081 10.2215/CJN.19611220 PMC8216620

[20] Narva, A. S., Norton, J. M. & Boulware, L. E. Educating patients about CKD: the path to self-management and patient-centered care. *Clin. J. Am. Soc. Nephrol.* **11**, 694–703 (2016). 26536899 10.2215/CJN.07680715 PMC4822666

[21] Tuot, D. S. et al. Variation in patients’ awareness of CKD according to how they are asked. *Clin. J. Am. Soc. Nephrol.* **11**, 1566–1573 (2016). 27340288 10.2215/CJN.00490116 PMC5012470

[22] Devraj, R., Borrego, M. E., Vilay, A. M., Pailden, J. & Horowitz, B. Awareness, self-management behaviors, health literacy and kidney function relationships in specialty practice. *World J. Nephrol.* **7**, 41–50 (2018). 29359119 10.5527/wjn.v7.i1.41 PMC5760511

[23] Wilkinson, T. J., Memory, K., Lightfoot, C. J., Palmer, J. & Smith, A. C. Determinants of patient activation and its association with cardiovascular disease risk in chronic kidney disease: a cross-sectional study. *Health Expect.* **24**, 843–852 (2021). 33835670 10.1111/hex.13225 PMC8235879

[24] Magadi, W. et al. Patient activation and its association with symptom burden and quality of life across the spectrum of chronic kidney disease stages in England. *BMC Nephrol.* **23**, 45 (2022). 35081904 10.1186/s12882-022-02679-w PMC8793272

[25] Johnson, M. L. et al. Patient activation with knowledge, self-management and confidence in chronic kidney disease. *J. Ren. Care* **42**, 15–22 (2016). 26537188 10.1111/jorc.12142

[26] Vélez-Bermúdez, M., Christensen, A. J., Kinner, E. M., Roche, A. I. & Fraer, M. Exploring the relationship between patient activation, treatment satisfaction, and decisional conflict in patients approaching end-stage renal disease. *Ann. Behav. Med.* **53**, 816–826 (2019). 10.1093/abm/kay091 PMC6937620 30535065

[27] Gair, R. M. et al. *Transforming Participation In Chronic Kidney Disease: Programme Report* (Renal Association, 2019).

[28] Greenwood, S. A. et al. Evaluating the effect of a digital health intervention to enhance physical activity in people with chronic kidney disease (Kidney BEAM): a multicentre, randomised controlled trial in the UK. *The Lancet Digital Health,* **6**, e23–e32 (2023). 10.1016/S2589-7500(23)00204-2 37968170

[29] Barker, F., Atkins, L. & de Lusignan, S. Applying the COM-B behaviour model and behaviour change wheel to develop an intervention to improve hearing-aid use in adult auditory rehabilitation. *Int. J. Audiol.* **55**, S90–S98 (2016). 27420547 10.3109/14992027.2015.1120894

[30] O’Connor, S. et al. Understanding factors affecting patient and public engagement and recruitment to digital health interventions: a systematic review of qualitative studies. *BMC Med. Inform. Decis. Mak.* **16**, 120 (2016). 27630020 10.1186/s12911-016-0359-3 PMC5024516

[31] Smekal, M. D. et al. Enhancing primary care capacity in chronic kidney disease management: a quality improvement educational initiative. *BMJ Open* **11**, e046068 (2021). 34753751 10.1136/bmjopen-2020-046068 PMC8578991

[32] Shlipak, M. G. et al. The case for early identification and intervention of chronic kidney disease: conclusions from a Kidney Disease: Improving Global Outcomes (KDIGO) Controversies Conference. *Kidney Int.* **99**, 34–47 (2021). 33127436 10.1016/j.kint.2020.10.012

[33] NHS England & NHS Improvement. *The Interface Between Primary And Secondary Care: Key Messages For NHS Clinicians And Managers* (NHS, 2017).

[34] Mallinckrodt, C. H., Watkin, J. G., Molenberghs, G. & Carroll, R. J. Choice of the primary analysis in longitudinal clinical trials. *Pharm. Stat.* **3**, 161–169 (2004).

[35] Siddiqui, O., Hung, H. M. J. & O’Neill, R. MMRM vs. LOCF: a comprehensive comparison based on simulation study and 25 NDA datasets. *J. Biopharm. Stat.* **19**, 227–246 (2009). 19212876 10.1080/10543400802609797

[36] Fitzmaurice, G. M., Laird, N. M. & Ware, J. H. *Applied Longitudinal Analysis* (John Wiley & Sons, 2004).

[37] Dinh, P. & Yang, P. Handling baselines in repeated measures analyses with missing data at random. *J. Biopharm. Stat.* **21**, 326–341 (2011). 21391005 10.1080/10543406.2011.550113

[38] Lightfoot, C. J. et al. Improving self-management behaviour through a digital lifestyle intervention: an internal pilot study. *J. Ren. Care* **50**, 283–296 (2024). 38296833 10.1111/jorc.12488

[39] Kanu, C., Brown, C., Barner, J., Chapman, C. & Walker, H. The effect of a tailored patient activation intervention in inflammatory bowel disease patients. *J. Contemp. Pharm. Pract.* **66**, 11–21 (2020).

[40] Schulz, K. F., Altman, D. G. & Moher, D. CONSORT 2010 statement: updated guidelines for reporting parallel group randomised trials. *PLoS Med.* **7**, e1000251 (2010). 20352064 10.1371/journal.pmed.1000251 PMC2844794

[41] Hadjiconstantinou, M. et al. Using Intervention Mapping to Develop a Digital Self-Management Program for People With Type 2 Diabetes: Tutorial on MyDESMOND. *J. Med. Internet Res.* **22**, e17316 (2020). 32391797 10.2196/17316 PMC7248797

[42] Davies, M. J. et al. Effectiveness of the diabetes education and self management for ongoing and newly diagnosed (DESMOND) programme for people with newly diagnosed type 2 diabetes: cluster randomised controlled trial. *BMJ* **336**, 491–495 (2008). 18276664 10.1136/bmj.39474.922025.BE PMC2258400

[43] Corbin, J. M. & Strauss, A. *Unending Work And Care: Managing Chronic Illness At Home* (Jossey-Bass, 1988).

[44] Michie, S., van Stralen, M. M. & West, R. The behaviour change wheel: a new method for characterising and designing behaviour change interventions. *Implement. Sci.* **6**, 42 (2011). 21513547 10.1186/1748-5908-6-42 PMC3096582

[45] Michie, S. et al. The behavior change technique taxonomy (v1) of 93 hierarchically clustered techniques: building an international consensus for the reporting of behavior change interventions. *Ann. Behav. Med.* **46**, 81–95 (2013). 23512568 10.1007/s12160-013-9486-6

[46] Schwarzer, R. *Self-efficacy: Thought Control Of Action*. p. 217-243 (Hemisphere Publishing Corp, 1992).

[47] Leventhal, H., Meyer, D. & Nerenz, D. *Medical Psychology* (S., RACHMAN, 1980).

[48] Bandura, A. Social cognitive theory of self-regulation. *Organ Behav. Hum. Decis. Process* **50**, 248–287 (1991).

[49] Hibbard, J. H., Mahoney, E. R., Stockard, J. & Tusler, M. Development and testing of a short form of the patient activation measure. *Health Serv. Res.* **40**, 1918–1930 (2005). 16336556 10.1111/j.1475-6773.2005.00438.x PMC1361231

[50] Devraj, R. & Wallace, L. S. Application of the content expert process to develop a clinically useful low-literacy Chronic Kidney Disease Self-Management Knowledge Tool (CKD-SMKT). *Res. Soc. Adm. Pharm.* **9**, 633–639 (2013). 10.1016/j.sapharm.2012.09.006 23182151

[51] Brown, S. A. et al. Kidney symptom questionnaire: development, content validation and relationship with quality of life. *J. Renal Care,* **44**, 162–173 (2018). 10.1111/jorc.12247 29797783

[52] Malmstrom, T. K., Miller, D. K., Simonsick, E. M., Ferrucci, L. & Morley, J. E. SARC-F: a symptom score to predict persons with sarcopenia at risk for poor functional outcomes. *J. Cachexia Sarcopenia Muscle* **7**, 28–36 (2016). 27066316 10.1002/jcsm.12048 PMC4799853

[53] Ahmad, S. et al. Evaluation of reliability and validity of the General Practice Physical Activity Questionnaire (GPPAQ) in 60–74 year old primary care patients. *BMC Fam. Pract.* **16**, 113 (2015). 26329981 10.1186/s12875-015-0324-8 PMC4557746

[54] Wilkinson, T. J., Palmer, J., Gore, E. F. & Smith, A. C. The validity of the ‘General Practice Physical Activity Questionnaire’ against accelerometery in patients with chronic kidney disease. *Physiother. Theory Pract*. **38**, 1–10 (2020). 10.1080/09593985.2020.1855684 33263260

[55] England, C. Y., Thompson, J. L., Jago, R., Cooper, A. R. & Andrews, R. C. Development of a brief, reliable and valid diet assessment tool for impaired glucose tolerance and diabetes: the UK Diabetes and Diet Questionnaire. *Public Health Nutr.* **20**, 191–199 (2017). 27609314 10.1017/S1368980016002275 PMC5244439

[56] Chan, A. H. Y., Horne, R., Hankins, M. & Chisari, C. The Medication Adherence Report Scale: a measurement tool for eliciting patients’ reports of nonadherence. *Br. J. Clin. Pharmacol.* **86**, 1281–1288 (2020). 31823381 10.1111/bcp.14193 PMC7319010

[57] Al-Jabi, S. W. et al. Depression in patients treated with haemodialysis: a cross-sectional study. *Lancet* **391**, 41 (2017). 29553441 10.1016/S0140-6736(18)30407-0

[58] Kieser, M. & Friede, T. Re-calculating the sample size in internal pilot study designs with control of the type I error rate. *Stat. Med.* **19**, 901–911 (2000). 10750058 10.1002/(sici)1097-0258(20000415)19:7<901::aid-sim405>3.0.co;2-l

[59] Harden, M. & Friede, T. Sample size recalculation in multicenter randomized controlled clinical trials based on noncomparative data. *Biom. J.* **62**, 1284–1299 (2020). 32128868 10.1002/bimj.201900138

[60] Wittes, J. & Brittain, E. The role of internal pilot studies in increasing the efficiency of clinical trials. *Stat. Med* **9**, 65–71 (1990). 2345839 10.1002/sim.4780090113

[61] Friede, T. & Kieser, M. Sample size recalculation in internal pilot study designs: a review. *Biom. J.* **48**, 537–555 (2006). 16972704 10.1002/bimj.200510238

[62] Chin, R. & Lee, B. *Principles And Practice Of Clinical Trial Medicine*. p. 303–323 (Academic Press, 2008).

