# Supplementary material for: The effects of a digital health intervention on patient activation in chronic kidney disease
Source: NPJ Digit Med. 2024 Nov 12;7:318. doi: 10.1038/s41746-024-01296-1 (PMC11558007; doi:10.1038/s41746-024-01296-1)
Supplement: Supplementary file 1 — Supplementary Material [file 41746_2024_1296_MOESM1_ESM.docx]

**Supplementary Material**

**Table of contents**

**Supplementary Tables**

Supplementary Table 1. Participant characteristics of the intervention group of those included in complete case (CC) and per-protocol (PP) analyses

Supplementary Table 2. Participant characteristics of those who did and did not complete PAM-13 (primary outcome) at 20 weeks

Supplementary Table 3. Complete case changes in PAM-13 for low and high PAM

Supplementary Table 4. Per-Protocol changes in PAM-13 for low and PAM

Supplementary Table 5. Mixed model repeated measures changes in PAM-13

Supplementary Table 6. CONSORT 2010 checklist

Supplementary Table 7. Protocol amendments to study design for definitive trial made to improve the delivery of MK&M and SMILE-K trial

**Supplementary Material Figures**

Supplementary Figure 1. Sankey diagram of PAM-13 level changes across groups

**Supplementary Material Notes**

Supplementary Note 1. Details of secondary outcome measures

Supplementary Note 2. Statistical analysis plan

**Supplementary Material References**

Supplementary References

**Supplementary Tables**

**Supplementary Table 1. Participant characteristics of the intervention group of those included in complete case (CC) and per-protocol (PP) analyses**

|  | **Intervention group participants included in CC analysis** | **Intervention group participants included in PP analysis** |
| --- | --- | --- |
| n | 280 | 210 |
| Baseline PAM score | 61.78 (±14.85) | 62.55 (±14.33) |
| Age | 59.39 (±13.64) | 59.25 (±13.54) |
| Sex, male n (%) | 161 (58%) | 117 (56%) |
| eGFR | 38.39 (±17.09) | 37.04 (±15.60) |

**Supplementary Table 2. Participant characteristics of those who did and did not complete PAM-13 (primary outcome) at 20 weeks**

|  | **Participants who completed PAM-13** | **Participants who did not complete PAM-13** |
| --- | --- | --- |
| n | 224 | 196 |
| Baseline PAM score | 62.85 (±14.19) | 60.78 (±15.56) |
| Age | 61.21 (12.69) | 58.23 (±13.99) |
| Sex, male n (%) | 131 (59%) | 119 (61%) |
| eGFR | 39.50 (±19.63) | 37.89 (±17.69) |

**Supplementary Table 3. Complete case changes in PAM-13 for low and high PAM**

|  | **Number of participants** | | **Mean change from baseline (95% CI)** | | **Mean intervention effect (95% CI)*** | **P value** |
| --- | --- | --- | --- | --- | --- | --- |
|  | **Control** | **Intervention** | **Control** | **Intervention** |  |  |
| **Low baseline PAM-13** |  |  |  |  |  |  |
| Week 10 | 40 | 53 | 7.26 (95% CI: 3.66 to 10.87) | 7.77 (95% CI: 4.64 to 10.90) | 0.51 (95% CI: -4.27 to 5.28) | 0.833 |
| Week 20 | 28 | 42 | 2.73 (95% CI: -1.41 to 6.86) | 9.33 (95% CI: 5.96 to 12.70) | 6.60 (95% CI: 1.27 to 11.94) | **0.016** |
|  |  |  |  |  |  |  |
| **High baseline PAM-13** |  |  |  |  |  |  |
| Week 10 | 74 | 115 | -1.29 (95% CI: -4.22 to 1.64) | 0.51 (95% CI: -1.84 to 2.86) | 1.80 (95% CI: -1.96 to 5.56) | 0.346 |
| Week 20 | 59 | 95 | 0.35 (95% CI: -2.92 to 3.62) | 1.73 (95% CI: -0.84 to 4.31) | 1.38 (95% CI: -2.78 to 5.55) | 0.513 |

_*_ _Intervention value minus control value, adjusted for baseline value and age_

**Supplementary Table 4. Per-Protocol changes in PAM-13 for low and PAM**

|  | **Number of participants** | | **Mean change from baseline (95% CI)** | | **Mean intervention effect (95% CI)*** | **P value** |
| --- | --- | --- | --- | --- | --- | --- |
|  | **Control** | **Intervention** | **Control** | **Intervention** |  |  |
| **Low baseline PAM-13** |  |  |  |  |  |  |
| Week 10 | 40 | 37 | 7.25 (95% CI: 3.70 to 10.80) | 8.16 (95% CI: 4.47 to 11.86) | 0.91 (95% CI: -4.23 to 6.05) | 0.725 |
| Week 20 | 28 | 30 | 2.73 (95% CI: -1.08 to 6.54) | 12.00 (95% CI: 8.32 to 15.68) | 9.17 (95% CI: 3.98 to 14.57) | **<0.001** |
|  |  |  |  |  |  |  |
| **High baseline PAM-13** |  |  |  |  |  |  |
| Week 10 | 74 | 86 | -1.26 (95% CI: -4.22 to 1.70) | 1.12 (95% CI: -1.63 to 3.86) | 2.38 (95% CI: -1.66 to 6.42) | 0.246 |
| Week 20 | 59 | 75 | 0.29 (95% CI: -2.93 to 3.50) | 1.33 (95% CI: -1.52 to 4.17) | 1.04 (95% CI: -3.26 to 5.34) | 0.633 |

_*_ _Intervention value minus control value, adjusted for baseline value and age_

**Supplementary Table 5. Mixed model repeated measures changes in PAM-13**

|  | **Control** | | **Intervention** | | **Difference (contrast estimate)** | **P*** |
| --- | --- | --- | --- | --- | --- | --- |
|  | Estimated mean change (95%CI) | SE | Estimated mean change (95%CI) | SE |  |  |
|  |  |  |  |  |  |  |
| **PAM-13** | 1.11 (95% CI: -1.44 to 3.66) | 1.30 | 4.10 (95% CI: 2.07 to 6.13) | 1.03 | 2.98 (95% CI: -0.28 to 6.24) | 0.073 |

_Data presented as mean and 95% confidence interval (CI), and standard error (SE). Data are absolute differences between estimated mean changes._

_P value shows differences, significance recognised as P<0.05_


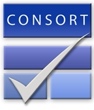
**Supplementary Table 6. CONSORT 2010 checklist**

CONSORT 2010 checklist of information to include when reporting a randomised trial*

| Section/Topic | Item No | Checklist item | Reported on page No |
| --- | --- | --- | --- |
| Title and abstract | | | |
|  | 1a | Identification as a randomised trial in the title | 1 |
|  | 1b | Structured summary of trial design, methods, results, and conclusions (for specific guidance see CONSORT for abstracts) | 4 |
| Introduction | | | |
| Background and objectives | 2a | Scientific background and explanation of rationale | 5 |
|  | 2b | Specific objectives or hypotheses | 5 |
| Methods | | | |
| Trial design | 3a | Description of trial design (such as parallel, factorial) including allocation ratio | 6 |
|  | 3b | Important changes to methods after trial commencement (such as eligibility criteria), with reasons | 6 |
| Participants | 4a | Eligibility criteria for participants | 6 |
|  | 4b | Settings and locations where the data were collected | 6 |
| Interventions | 5 | The interventions for each group with sufficient details to allow replication, including how and when they were actually administered | 7 |
| Outcomes | 6a | Completely defined pre-specified primary and secondary outcome measures, including how and when they were assessed | 8 |
|  | 6b | Any changes to trial outcomes after the trial commenced, with reasons | N/A |
| Sample size | 7a | How sample size was determined | 8 |
|  | 7b | When applicable, explanation of any interim analyses and stopping guidelines | 9 |
| Randomisation: |  |  |  |
| Sequence generation | 8a | Method used to generate the random allocation sequence | 6 |
|  | 8b | Type of randomisation; details of any restriction (such as blocking and block size) | 6 |
| Allocation concealment mechanism | 9 | Mechanism used to implement the random allocation sequence (such as sequentially numbered containers), describing any steps taken to conceal the sequence until interventions were assigned | 6 |
| Implementation | 10 | Who generated the random allocation sequence, who enrolled participants, and who assigned participants to interventions | 6 |
| Blinding | 11a | If done, who was blinded after assignment to interventions (for example, participants, care providers, those assessing outcomes) and how | 7 |
|  | 11b | If relevant, description of the similarity of interventions | N/A |
| Statistical methods | 12a | Statistical methods used to compare groups for primary and secondary outcomes | 9 |
|  | 12b | Methods for additional analyses, such as subgroup analyses and adjusted analyses | 9 |
| Results | | | |
| Participant flow (a diagram is strongly recommended) | 13a | For each group, the numbers of participants who were randomly assigned, received intended treatment, and were analysed for the primary outcome | 10 |
|  | 13b | For each group, losses and exclusions after randomisation, together with reasons | 10 |
| Recruitment | 14a | Dates defining the periods of recruitment and follow-up | 6 |
|  | 14b | Why the trial ended or was stopped | 10 |
| Baseline data | 15 | A table showing baseline demographic and clinical characteristics for each group | 10 |
| Numbers analysed | 16 | For each group, number of participants (denominator) included in each analysis and whether the analysis was by original assigned groups | 10 |
| Outcomes and estimation | 17a | For each primary and secondary outcome, results for each group, and the estimated effect size and its precision (such as 95% confidence interval) | 10-13 |
|  | 17b | For binary outcomes, presentation of both absolute and relative effect sizes is recommended | N/A |
| Ancillary analyses | 18 | Results of any other analyses performed, including subgroup analyses and adjusted analyses, distinguishing pre-specified from exploratory | 10-13 |
| Harms | 19 | All important harms or unintended effects in each group (for specific guidance see CONSORT for harms) | 10 |
| Discussion | | | |
| Limitations | 20 | Trial limitations, addressing sources of potential bias, imprecision, and, if relevant, multiplicity of analyses | 16-17 |
| Generalisability | 21 | Generalisability (external validity, applicability) of the trial findings | 14-17 |
| Interpretation | 22 | Interpretation consistent with results, balancing benefits and harms, and considering other relevant evidence | 14-17 |
| Other information | | |  |
| Registration | 23 | Registration number and name of trial registry | 5 |
| Protocol | 24 | Where the full trial protocol can be accessed, if available | 5 |
| Funding | 25 | Sources of funding and other support (such as supply of drugs), role of funders | 2 |

Citation: Schulz KF, Altman DG, Moher D, for the CONSORT Group. CONSORT 2010 Statement: updated guidelines for reporting parallel group randomised trials. BMC Medicine. 2010;8:18.
© 2010 Schulz et al. This is an Open Access article distributed under the terms of the Creative Commons Attribution License (<http://creativecommons.org/licenses/by/2.0>), which permits unrestricted use, distribution, and reproduction in any medium, provided the original work is properly cited.

*We strongly recommend reading this statement in conjunction with the CONSORT 2010 Explanation and Elaboration for important clarifications on all the items. If relevant, we also recommend reading CONSORT extensions for cluster randomised trials, non-inferiority and equivalence trials, non-pharmacological treatments, herbal interventions, and pragmatic trials. Additional extensions are forthcoming: for those and for up-to-date references relevant to this checklist, see [www.consort-statement.org](http://www.consort-statement.org).

**Supplementary Table 7. Protocol amendments to study design for definitive trial made to improve the delivery of MK&M and SMILE-K trial**

| **Amendment** | **Detail** |
| --- | --- |
| Inclusion criteria | The inclusion criteria were broadened to provide a larger population sample from which to recruit and include patients with confirmed progressive kidney *or* eGFR of 15-59 ml/min/1.73m^2^. |
| Participant information sheet | The participant information sheet was extensive in its current state. It was decided to split the document into two: one with main study information and another with data protection information. |
| Outcome assessment | An additional reminder email was developed and sent to participants who had not completed the survey within 5 days. This timeframe was based on the mean time taken to complete and return the survey from the pilot data and suggestions from interviewed participants. |
| Power calculation | Using previously published methodology for re-estimating sample sizes from internal pilot data(Wittes and Brittain, 1990), a re-calculation yielded a revised minimum sample size of 280 (β=0.80 and α=0.050). Assuming a 40% dropout (rate from internal pilot study was 38%), this provided a final required sample size of 392. |

NB. Confirmed progressive kidney disease refers to a diagnosed kidney disease where eGFR ≥60 ml/min/1.73m2 and were assessed by their nephrologist as having a kidney condition that was likely to progress, either because of the nature of their underlying kidney disease (e.g. polycystic kidney disease, relapsing glomerulonephritis), have high levels of proteinuria, or have co-morbidities that made risk of progression more likely (e.g. difficult to control blood pressure or reno-vascular disease). Whilst an inability to speak English was not an exclusion criterion, all communication, study information, and MK&M, were provided in English, and thus individuals who could not speak, read, and/or write English were likely unable to participate.

Full protocol has been previously published (16).

**Supplementary Material Figures**

**Supplementary Figure 1. Sankey diagram of PAM-13 level changes across groups**

|  |
| --- |
| 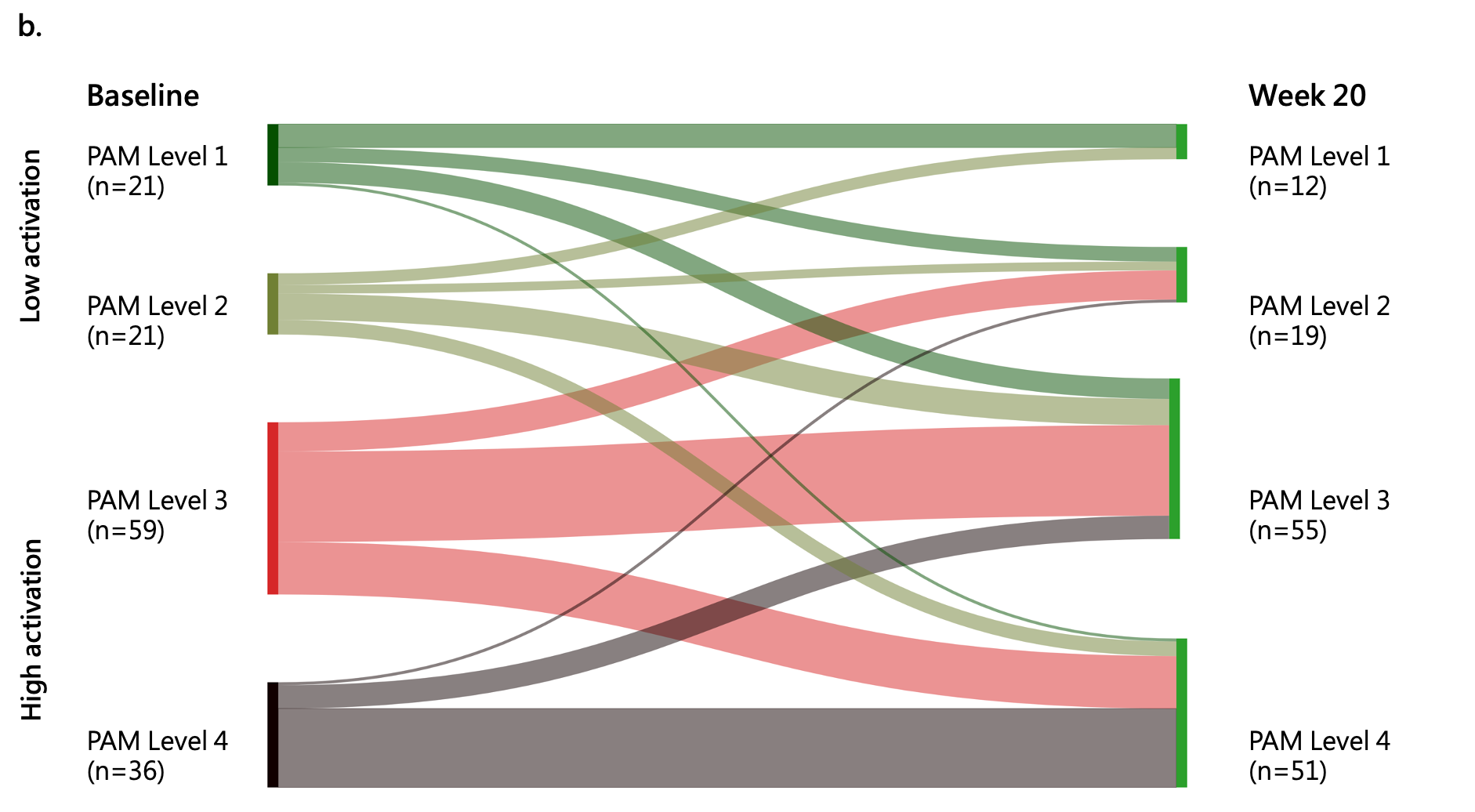 |
| **Figure 1.** **Sankey diagram of PAM-13 level changes across groups**  The diagram shows the flow of participants from baseline to week 20 in regard to their PAM-13 level in each group  a, control group changes in PAM-13 levels  b, intervention group changes in PAM-13 levels  The thickness of each node represents the number of participants |

**Supplementary Material Notes**

**Supplementary Note 1. Details of secondary outcome measures**

**Chronic Kidney Disease Self-Management Knowledge Tool (CKD-SMKT)**

Chronic Kidney Disease Self-Management Knowledge Tool (CKD-SMKT)(1) assesses kidney disease knowledge and related self-management behaviours. The questionnaire comprises of ten statements of self-management behaviours to which the respondent must indicate if they believe this is true, false or I do not know, and if they have done this in the last six months (yes or no). Knowledge of CKD and self-management behaviours was calculated as a percentage of correct responses to the 10-item CKD-SMKT. Participants indicated past performance of behaviours using a yes/no response. Awareness of kidney health was assessed on 5-point Likert scale from “I know nothing” to “I know everything I need to know”. Lower scores indicate greater awareness of kidney health awareness.

**Kidney Symptom Questionnaire (KSQ)**

The KSQ is a questionnaire to assess the frequency, intrusiveness, and total impact of a range of 11 symptoms (itching, sleep disturbance, loss of appetite, excessive tiredness, pain in bones/joints, poor concentration/mental alertness, impotence (optional question), loss of muscle strength/power, shortness of breath muscle spasm/stiffness, restless legs). This questionnaire has recently been validated by our group (2).

**Medication Adherence Report Scale (MARS-5)**

The MARS-5 is a non-disease specific questionnaire used to measure adherence to medications and has been previously used in CKD (3, 4). This comprises of five questions regarding changing medication dosage, forgetting to take medication, consciously stopping taking medication, skipping medication and using less than prescribed. Participants are asked to indicate the frequency that they exhibit these adherence behaviours on a five-point Likert scale from ‘always’ to ‘never’. For each question, scores ascend from one point (always) to five points (never) which are then aggregated to give the final score. A total score of less than 25 points indicates low adherence to medication.

**UK Diabetes and Diet Questionnaire (UKDDQ)**

UK Diabetes and Diet Questionnaire (UKDDQ)(5) asks respondents how often they consumed certain foods (vegetables, fruits, sugary drinks, processed meat) over the last month on a 6-point Likert scale. Items are scored on the frequency of consumption ranging from 5 (healthiest) to 0 (least healthy) choice and classified into “healthy” (4&5), “less healthy” (2&3) and “unhealthy” (0&1) choices. The total UKDDQ score was calculated from the 20 items, giving a final score ranging from 0 to 100. The mean UKDDQ score was also calculated from the 20 items, giving a final score ranging from 0 to 5. A higher score indicates a ‘healthier’ diet. Number (%) of participants achieving ‘healthy’ scores was recorded, alongside the number of healthy, less healthy, and unhealthy food choices.

**General Practice Physical Activity Questionnaire (GPPAQ)**

The GPPAQ was developed by the World Health Organization and Department of Health to provide a simple one-page questionnaire detailing a 4-level Physical Activity Index (PAI) reflecting an individual's current physical activity (i.e. Active, Moderately Active, Moderately Inactive, Inactive) (6). Participants were categorised into ‘Active’ and ‘Inactive’

**SARC-F questionnaire**

Recommended by the EWGSOP2 (7), the SARC‐F questionnaire includes five components: Strength, Assistance walking, Rise from a chair, Climb stairs, and Falls. SARC‐F items were selected to reflect health status changes associated with the consequences of sarcopenia (8). SARC‐F scale scores range from 0 to 10 (i.e. 0–2 points for each component; 0 = best to 10 = worst) and a score equal to or greater than 4 is predictive of sarcopenia (8).

**Sit-to-stand-60 second test (STS-60)**

In order to capture a form of objective physical function, at the end of the Part A online survey, we will ask participants to complete a 60-second sit-to-stand test. In this test participants will be asked to record how many sit-to-stand cycles they can complete with their hands across their chest.

**Supplementary Note 2. Statistical analysis plan**

**STATISTICAL ANALYSIS PLAN (SAP)**

**Study Title:**  SMILE-K: ‘Self-Management Intervention through Lifestyle Education for Kidney health’

**Short Title:** SMILE-K

**Funder:** Stoneygate Trust and the Leicester NIHR Biomedical Research Centre

**Sponsor:** University of Leicester

**Protocol Version:**  2.0 Date: 05/12/2021

**SAP Version:** 2.0 Date: 01/02/2022

**Prepared by:** Dr Courtney Lightfoot

Research Associate, Clinical Trial Facilitator, University of Leicester

**Approved by:** Professor Alice Smith

Professor of Lifestyle Medicine, University of Leicester

Dr Clare Gillies

Associate Professor in Medical Statistics, University of Leicester, Leicester Real World Evidence Unit

Professor Thomas Yates

Professor of Physical Activity, Sedentary Behaviour and Health, Diabetes Research Centre, University of Leicester, NIHR Leicester Biomedical Research Centre

Dr Matthew Graham-Brown

Associate Professor of Renal Medicine, University of Leicester, and Honorary Consultant Nephrologist, University Hospitals of Leicester

Dr Thomas Wilkinson

Research Fellow, University of Leicester, NIHR Leicester Biomedical Research Centre

1. **Introduction**

This Statistical Analysis Plan (SAP) describes the planned analysis and reporting for the Self-Management Intervention through Lifestyle Education for Kidney health (SMILE-K) trial, which assesses the effect of an evidence-based and theory-based digital self-management programme ‘My Kidneys & Me’ (MK&M) on patient activation and related self-management behaviours in people with non-dialysis CKD. All work planned and reported for this SAP will follow internationally accepted guidelines where relevant. The reader of this SAP is encouraged to also read the trial protocol (V2.0 05.12.2021) and published protocol (<https://pubmed.ncbi.nlm.nih.gov/36385018/>).

The purpose of this SAP is to outline the planned analyses that will be performed on the data at the end of the trial. The SAP will be amended if there are any substantial changes to planned analyses and will be finalised before the database lock.

**1.1 Study Background**

Many people living with chronic kidney disease (CKD) are expected to self-manage their condition. Patient activation is the term given to describe the knowledge, skills and confidence a person has in managing their health and is closely related to engagement in preventive health behaviours. Self-management interventions have the potential to improve remote disease management and health outcomes. We are testing an evidence-based and theory-based digital self-management structured 10-week programme developed for people with CKD called ‘My Kidneys & Me’ (MK&M). The primary aim of the study (Self-Management Intervention through Lifestyle Education for Kidney health (SMILE-K)) is to assess the effect on patient activation levels.

1. **Study Objectives**
   1. **Primary Objectives**

The primary objective of this study was to assess the effect of MK&M on patient activation.

- 1. **Secondary Objectives**

In addition, secondary objectives will investigate the effect of MK&M on CKD-related self-management behaviours:

- CKD self-management knowledge and behaviour
- Kidney health awareness
- Dietary choices and behaviours
- Symptoms
- Medication adherence
- Physical activity
- Physical function

Other exploratory outcome measures include uptake and engagement of MK&M, healthcare usage, quality of life, illness perception, and sarcopenia. In addition, patient experience of participating in the SMILE-K trial and using MK&M will be explored.

Hypotheses:

1. My Kidneys & Me leads to improvements in patient activation

2. My Kidneys & Me leads to improvements in CKD-related self-management behaviours

1. **Study Design**
   1. **Overview**

This study is a single-blind longitudinal randomised controlled trial with a nested pilot study. The nested pilot study will assess the feasibility of the intervention and study design before continuation to a full RCT is considered. Participants will be randomised, using 2:1 randomisation, to receive the intervention of MK&M or to standard of care (control group). The study intervention period will last 20 weeks with outcome measures assessed at baseline (preintervention), week 10 (interim), and week 20 (postintervention).

- 1. **Participants**

Participants are CKD patients, aged 18 years or older, with CKD stages 3-4 (estimated glomerular filtration rate, eGFR 15-59ml/min/1.73m^2^) or diagnosed with a progressive kidney condition with an eGFR >60ml/min/1.73m^2^ (e.g., polycystic kidney disease) not receiving renal replacement therapy. They will be recruited from kidney hospital services across England.

- - 1. **Intervention arm**

Participants randomised to the intervention group will receive immediate access to the MK&M program with a unique user login. The MK&M programme forms part of the award-winning and quality-assured MyDESMOND e-learning platform. The educational sessions provide information about the kidneys, CKD, its treatment and the different ways to self-manage. ‘How to’ booster sessions are interactive educational sessions, which provide instructions on how to perform self-management behaviours and are released weekly. The health tracker features allow patients to self-monitor different aspects of their health that are involved in the self-management of the condition: body weight and measurements, fruit and vegetable intake, symptoms, smoking, cholesterol and blood pressure. Similarly, the ‘Activity’ feature allows patients to track their physical activity. The ‘Decision maker’ feature is a tool to help patients create and monitor their own health-related goals, as this works through a series of questions to help patients identify which goals are most important to them and how they can achieve these by overcoming identified barriers.

- - 1. **Control arm**

Participants in the control arm will receive usual care and will be asked to follow their clinical care plans. All participants will receive access to MK&M following study completion.

- - 1. **Sample size**

An initial sample size of N=432 participants, including dropouts, was estimated to detect a clinically important difference of 4-points in the PAM-13. Using data from an internal pilot (pooled variance at 10-weeks: 283.798; estimated difference: 6.3) was used to review the sample size accordingly, this review yielded a revised minimum sample size of 151 (β=0.90 and α=0.050). Assuming an estimated 40% dropout (rate from internal pilot study was 38%), this provided a revised sample size of 211 (n=141 in the intervention group, and n=70 in the control group). Data from the full RCT will be used to identify an MCID for the PAM-13 in CKD using if feasible, thus recruitment will continue until December 2022.

- - 1. **Randomisation and blinding**

The trial design is a randomised controlled trial. Participants will be randomised into two groups – the MK&M group and control group. Randomisation will be performed by the research team in a single-blind fashion using a computer-generated program. Patients will be stratified based on age (≤63, >63 years) to ensure comparatively equal representative age characteristics in both groups. These values are based on the median attained from preliminary unpublished data from 120 patients with an eGFR <60 ml/min/m^2^ in two ongoing observational studies by our group.

1. **Statistical Analysis Plan (SAP)**
   1. **SAP objectives**

The objective of this SAP is to describe the statistical analyses to be carried out for the final analysis of SMILE-K.

- 1. **General principles**

All analyses will be complete case and will be reported by treatment. Analyses were conducted using both complete case (CC) (i.e., only participants with available outcome data) and per-protocol (PP) approaches on both the primary and secondary outcomes. A PP analysis will include all randomised patients who met the specific minimum criteria: activation of MK&M account and logging in more than once. Continuous variables will be summarised by mean and standard deviation (SD), median and interquartile range (IQR (n-n)). Categorical variables will be summarised by N (%). Change data will be presented as means and 95% confidence intervals.

- 1. **Study protocol**

The current study protocol at the time of writing is Version 2.0, dated 5^th^ December 2021.

- 1. **Deviations to those specified in the protocol**

The following measures specified in the protocol were not available at the time of the final analysis:

- Anthropometry: height, weight, body mass index, waist and hip circumference
- Muscle phenotyping: bioelectrical impendence analysis, ultrasonography
- Physical function: gait speed, handgrip strength, sit-to-stand-60, timed-up-and-go’ test
- Physical activity: accelerometery (7 days)
  1. **Software**

Quantitative analyses will be carried out using SPSS 28, or a higher version of the program.

Qualitative analyses will be carried out using thematic analysis. Data will be stored and managed in NVivo 12, or a higher version of the program.

1. **Analysis**

Full analysis set: randomised, not withdrawn or died before 20-week follow-up.

Analysis for each outcome will be based on the subjects within the full-analysis set population that also have data at the final visit for that outcome.

- 1. **Primary Outcome**
     1. **Definition and Derivation of Primary Outcome**

The primary outcome is the absolute change in patient activation at 20-weeks, which will be measured using the Patient Activation Measure (PAM-13).

- - 1. **Hypothesis to be investigated**

The null hypothesis for the primary analysis is that there is no difference in the primary outcome between the intervention and control arms across baseline and 20-week follow-up.

- 1. **Secondary Outcomes**

A number of secondary outcomes, listed below, will be investigated, all of which relate to general gastrointestinal health and quality of life.

- - 1. **Main secondary outcomes**

The following secondary outcomes are of particular interest and will be analysed using a relevant statistical model. Please note that this separation of secondary outcomes is informal and purely for analysis purposes.

- CKD self-management knowledge and behaviours, kidney health awareness: Chronic Kidney Disease Self-Management Knowledge Tool (CKD-SMKT)
- Symptoms: Kidney Symptom Questionnaire (KSQ)
- Sarcopenia: Strength, Assistance walking, Rising from a chair, Climbing stairs, and Falls (SARC-F)
- Physical activity: General Practice Physical Activity Questionnaire (GPPAQ)
- Diet: UK Diabetes and Diet Questionnaire (UKDDQ)
- Medication adherence: Medication Adherence Report Scale (MARS-5)
- Healthcare use: Modified Economic Patient Questionnaire (EPQ)
- Physical function: Sit-to-stand-60 (STS-60)
- Illness perception: IPQ-R
- Health status/ quality of life SF-12
  - 1. **Other secondary outcomes**

The remaining secondary outcomes, listed below, will be summarised.

- Uptake and usability of MK&M
- Patient experiences of MK&M
  - 1. **Hypotheses to be investigated**

The null hypothesis is that there is no difference in any of the secondary outcomes between the intervention and control arms across baseline and follow-up.

- 1. **Analysis of the primary outcome**

The primary analysis will be conducted using a CC approach. This includes all randomised participants except those with missing outcome data at 20-weeks, who will be excluded from the analysis. This analysis will follow the modified (given CC approach) intention-to-treat principle, with participants being analysed based on the arm to which they were randomised. Treatment effects (change final-baseline) on primary outcome will be analysed using generalised linear regression models to determine between-group differences, adjusted for age and baseline values, with change in outcome score as the dependent variable and group assignment as covariates. The effect will also be estimated using a PP analysis, which will only include participants who were compliant with the protocol (defined as those who activated their MK&M account and logged in more than once).

Additional post-hoc analysis will be performed to determine if differences exist in the changes of the primary outcome (PAM-13 score) between those with low and high PAM scores. Whether activation level modified the treatment effect was determined by the F-test significance when entered as an interaction in a generalised linear model, adjusting for age and baseline value.

- 1. **Analysis of the secondary outcomes**

Secondary outcomes will use the same analysis methods as the primary analysis.

- 1. **Sensitivity analysis**

A sensitivity analysis will be conducted using a Mixed Model for Repeated Measures (MMRM) to account for missing data under a rising at random assumption. For the MMRM analysis, the independent variables of the analysis model will include group (intervention or control), timepoint (10 or 20 weeks), group by timepoint interaction, PAM-13 score at baseline, and age as fixed effects. An unstructured covariance matrix for measurements within the same participant will be used.

1. **Tables and figures**

The layout of the tables and figures will be agreed based on a report using dummy treatment codes prior to database lock. Approval of the format of this report will be documented separately.

1. **Listings**

Listings will be provided for all adverse events.

**Supplementary Material References**

**Supplementary References**

1. Devraj R, Wallace LS. Application of the content expert process to develop a clinically useful low-literacy Chronic Kidney Disease Self-Management Knowledge Tool (CKD-SMKT). Research in Social and Administrative Pharmacy. 2013;9(5):633-9.

2. Brown SA TF, Clarke AL. Kidney Symptom Questionnaire: Development, content validation and relationship with quality of life. Journal of Renal Care. 2018.

3. Hjemås BJ, Bøvre K, Mathiesen L, Lindstrøm JC, Bjerknes K. Interventional study to improve adherence to phosphate binder treatment in dialysis patients. BMC nephrology. 2019;20(1):1-10.

4. Zimbudzi E, Lo C, Misso M, Ranasinha S, Zoungas S. Effectiveness of management models for facilitating self-management and patient outcomes in adults with diabetes and chronic kidney disease. Systematic reviews. 2015;4(1):81.

5. England CY, Thompson JL, Jago R, Cooper AR, Andrews RC. Development of a brief, reliable and valid diet assessment tool for impaired glucose tolerance and diabetes: the UK Diabetes and Diet Questionnaire. Public Health Nutr. 2017;20(2):191-9.

6. Ahmad S, Harris T, Limb E, Kerry S, Victor C, Ekelund U, et al. Evaluation of reliability and validity of the General Practice Physical Activity Questionnaire (GPPAQ) in 60–74 year old primary care patients. BMC family practice. 2015;16(1):113.

7. Cruz-Jentoft AJ, Bahat G, Bauer J, Boirie Y, Bruyère O, Cederholm T, et al. Sarcopenia: revised European consensus on definition and diagnosis. Age and ageing. 2018;48(1):16-31.

8. Malmstrom TK, Morley JE. SARC-F: a simple questionnaire to rapidly diagnose sarcopenia. Journal of the American Medical Directors Association. 2013;14(8):531-2.
